# The association of child maltreatment and systemic inflammation in adulthood: A systematic review

**DOI:** 10.1371/journal.pone.0243685

**Published:** 2021-04-08

**Authors:** Daniel M. Kerr, James McDonald, Helen Minnis

**Affiliations:** 1 Institute of Health and Wellbeing, University of Glasgow, Glasgow, United Kingdom; 2 NHS Greater Glasgow and Clyde, Glasgow, United Kingdom; Montana State University, UNITED STATES

## Abstract

**Introduction:**

Child maltreatment (CM) is associated with mental and physical health disorders in adulthood. Some studies have identified elevated markers of systemic inflammation in adult survivors of CM, and inflammation may mediate the association between CM and later health problems. However, there are methodological inconsistencies in studies of the association between CM and systemic inflammation and findings are conflicting. We performed a systematic review to examine the association of CM with systemic inflammation in adults.

**Methods:**

A pre-registered systematic review was performed following PRISMA guidelines. Medline, Embase, Scopus and PsychInfo were searched for studies of the association of CM with blood markers of inflammation in adults. Quality was assessed using the Crowe Critical Appraisal Tool. We had intended to perform a meta-analysis, but this was not possible due to variation in study design and reporting.

**Results:**

Forty-four articles met criteria for inclusion in the review. The most widely reported biomarkers were C-Reactive Protein (CRP) (n = 27), interleukin-6 (IL-6) (n = 24) and Tumour Necrosis Factor-alpha (TNF-a) (n = 17). Three studies were prospective (all relating to CRP) and the remainder were retrospective. 86% of studies were based in high income countries. In the prospective studies, CM was associated with elevated CRP in adulthood. Results of retrospective studies were conflicting. Methodological issues relating to the construct of CM, methods of analysis, and accounting for confounding or mediating variables (particularly Body Mass Index) may contribute to the uncertainty in the field.

**Conclusions:**

There is some robust evidence from prospective studies that CM is associated with elevated CRP in adulthood. We have identified significant methodological inconsistencies in the literature and have proposed measures that future researchers could employ to improve consistency across studies. Further prospective, longitudinal, research using robust and comparable measures of CM with careful consideration of confounding and mediating variables is required to bring clarity to this field.

## Introduction

Childhood maltreatment (CM) is common worldwide [1,2]. Studies have consistently shown CM, particularly multiple and cumulative exposures, to be associated with a range of adverse physical, psychological, and social outcomes [1–6]. That this association persists after adjustment for environmental and behavioural factors suggests underlying biological mechanisms which may mediate the relationship between CM and health and social outcomes in later life [2,7]. Understanding the biological correlates of CM will help to clarify the mechanisms linking CM with adverse outcomes, offers the prospect of enhanced risk stratification of young people who have been subject to maltreatment and may identify new treatment targets to break the link between childhood experiences and adverse physical and mental health outcomes in adulthood [2].

Low-grade systemic inflammation, has been proposed to be defined by a 2–3 fold elevations in inflammatory markers like C-reactive protein (CRP), Interleukin-6 (IL-6) and tumour necrosis factor alpha (TNF-a) [8]. This represents a chronic low-level activation of the immune system (likely representing excessive sensitivity to inflammatory stimuli and deficiencies of the anti-inflammatory pathways which would normally terminate such responses) and is distinguished from high-grade inflammatory states with markedly elevated inflammatory markers such as occurs in acute infections, severe illnesses, and auto-inflammatory diseases. Low-grade, systemic inflammation has been identified in adult survivors of CM [9].

### Inflammation and physical health disorders

Low-grade inflammation has been associated with a range of physical health conditions such as cardiovascular disease and diabetes [10,11]. Notably, a large body of work has associated low-grade elevations in CRP with cardiovascular events—however subsequent work has questioned the direction of causality in this relationship [12]. Other inflammatory markers have been associated with cardiovascular disease, particularly Interleukin-6 [10]. A large international study using Mendelian randomisation techniques has supported a causal relationship between elevated levels of IL-6 and cardiac disease [10]. Further supporting evidence for the role of low-grade inflammation in the causal pathway towards cardiovascular disease is provided by the recent CANTO trial of the specific IL-1b antagonist Canakinumab which was shown to reduce rates of further myocardial infarction (MI), stroke and death in patients with elevated CRP who were treated following infarction MI [13].

### Inflammation and mental health disorders

Low-grade inflammation is also associated with a range of mental health disorders. A wide body of work has associated major depressive disorder with low-grade elevations in inflammatory markers like CRP, IL-6, and TNF-a [14,15]. The neurobiological effects of peripheral cytokines may mediate the relationship between external stressors and depression [14]. Low-grade inflammation is also associated with conditions like post-traumatic stress disorder (PTSD), schizophrenia, and bipolar affective disorder [8,16], and with elevated risk of attempted suicide [15]. These associations are, however, complicated by bidirectional effects [17,18], and shared risk factors [18], however inflammation has been demonstrated to have direct effects on neurobiology and brain development which are likely contribute to the aetiology of mental health disorders such as depression and schizophrenia [19,20]. A recent Mendelian randomisation analysis has suggested a causal relationship between CRP and both schizophrenia and bipolar affective disorder [16]. There is also emerging evidence that anti-inflammatory drugs may be effective in treating major depressive disorder and that conventional anti-depressants may have anti-inflammatory actions [19,21].

### Inflammation and child maltreatment

An emerging body of evidence, therefore, has shown that low-grade systemic inflammation is associated with increased risk of physical and mental health disorders. Although it is known that CM is associated with low-grade, systemic inflammation [9], previous reviews have identified significant heterogeneity in the literature particularly in relation to the definition and ascertainment of CM [22,23]. Studies have offered varying definitions of CM ranging from narrowly focused childhood physical or sexual abuse, to more broadly defined Adverse Childhood Experience (ACEs) [22,23]. Differing patterns of CM will likely have different effects on development, contributing to the heterogeneity in the literature. Furthermore, research in this area has highlighted the role of potential mediators between CM and inflammation, particularly body mass index (BMI). Obesity is associated with elevated markers of peripheral inflammation such as CRP and IL-6 [24], and CM is associated with increased risk of obesity [25]. This raises questions about the causality of this relationship that were not fully addressed in previous reviews [22,23]. The most recent systematic review of the association of CM and inflammation in adults was conducted in 2016 and identified 25 eligible studies. There have been significant developments in the literature over the past 6 years [23]. The 2016 review included any form of childhood trauma (excluding socioeconomic status), included studies of participants with pro-inflammatory conditions such as cancer and was limited to studies reporting CRP, IL-6, or TNF-a [23]. More recently in 2020 Kuhlman and colleagues reported a meta-analysis of the association between early life adversity (ELA) and inflammation in under 18s [26]. This identified 27 relevant studies and found small associations between ELA and inflammation which only reached statistical significance for CRP. This included all forms of early adversity. In response to the existing literature we aimed to perform an updated systematic review of the association between CM and inflammation in adulthood. In an effort to reduce the significant heterogeneity in this field we aimed to limit our exposure to child abuse and neglect rather than wider forms of adversity.

## Methods

We performed a systematic review of the association between CM and low-grade inflammation. Preferred Reporting Items for Systematic Reviews and Meta-Analyses (PRISMA) guidelines were followed [27]. The search and synthesis plan were pre-specified in a protocol registered with PROSPERO (CRD42020187027).

### Research questions

Our primary research question was: “Is CM associated with elevated markers of systemic inflammation adulthood?”. We also pre-specified secondary questions: “Are differences in later life inflammation associated with specific sub-types, timings, or durations of abuse?” and “What mediates any association between CM and inflammation?”.

### Inclusion criteria

We included full text articles reporting non-randomised observational studies. Review articles and conference abstracts were not included. Our study population was human adults (>18 years of age). Participants could be drawn from healthy samples or clinical samples (with mental or physical health disorder), however we excluded studies of participants with pro-inflammatory physical health conditions, in particular autoimmune disease, and cancer. Participants could be drawn from community or hospital-based samples.

Our exposure was CM, defined as physical abuse, sexual abuse, emotional abuse, physical neglect and/or emotional neglect occurring at least once before the age of 18. We did not place any restrictions on how CM was recorded. Studies could use retrospective or prospective ascertainment; and could record CM using validated scales, or specific measures developed for their study if this was described. CM could be reported as an overall construct or broken down into sub-types of abuse and neglect. Studies could compare between a CM exposed group and a control group or utilise a continuous measure of CM in a sample. We excluded studies which exclusively reported wider adverse childhood experiences (eg. bullying, parental divorce, poverty etc.). Studies which included wider ACEs and abuse/neglect were included but only outcomes relating to abuse/neglect are reported.

Our outcome was blood levels of inflammatory markers measured in adulthood (>18). Any marker of the inflammatory response measured in the blood was eligible for inclusion. We excluded studies which reported on stimulated responses (e.g. to stress testing or biological stimulation), studies reporting exclusively on gene expression, *in vitro* production of inflammatory mediators, and studies exclusively measuring inflammation in the central nervous system (e.g. cerebrospinal fluid).

### Search strategy

We searched MedLine, Embase, PsychInfo, and Scopus. Our first search term aimed to capture CM. This included MeSH terms “child abuse”, “child abuse, sexual”, “adult survivors of child abuse”, “physical abuse”, “child, abandoned”, “adolescent, institutionalized”, “adult survivors of child adverse events” and “adverse childhood experiences”, supplemented by title and abstract searches for related terms. The second term sought to identify broadly defined inflammation. This included MeSH terms “inflammation”, “C-reactive protein”, “acute phase proteins”, “tumour necrosis factor alpha”, “interleukins”, “cytokines”, “immune system”, “fibrinogen”, “leukocytes”, and “lymphocytes”. This was supplemented by title and abstract searches for related terms.

These terms were combined using the Boolian operator “AND”, and duplicates were removed.

The search was adapted to utilise relevant keywords in the other databases used and full information is available in S1 File.

This search was supplemented by manual checking of reference lists of retrieved articles and checking the reference lists of previous reviews in this area.

### Methods of review

Records were initially screened against inclusion criteria by one reviewer, and a second reviewer independently reviewed a sub-sample of 25% of titles. All included articles were then reviewed by a second reviewer to confirm that they met inclusion criteria. Disagreements were resolved through conference with a third author. Inter-rater agreement was 94%.

Risk of bias assessment was performed at study level using the Crowe Critical Appraisal Tool (CCAT) v1.4 (https://conchra.com.au/wp-content/uploads/2015/12/CCAT-form-v1.4.pdf). This is a tool for assessment of risk of bias in non-randomised studies. Key components of risk of bias assessment include sampling, ascertainment of exposure, measurement of outcome, and statistical analysis including adjustment for relevant confounding variables. The CCAT assigns a total score from 0–40. According to the tools guidelines studies can be categorised as low quality (<20), moderate quality (20–29), and high quality (30+). All papers were rated by one reviewer, and the second reviewer independently quality rated a sub-sample of 25% of papers. Again, disagreements were resolved through conference with a third author. Inter-rater agreement was 90%.

Data extraction was performed using a pre-specified form including sample size, demographic variables, study setting, population type, measure of abuse, inflammatory marker measured, statistical, methods and key results. Data extraction was performed by one reviewer, with a second reviewer independently performing data extraction for a sample of 25% of included papers.

### Synthesis

We had intended to perform a meta-analysis of the most widely reported inflammatory markers as specified in the protocol. A more detailed review of included articles showed that this was not feasible due to differences in the construct of CM being utilised, incommensurable methods of analysis and inconsistent accounting for covariates. This is discussed further below. These challenges led us to conclude that a meta-analysis would be of questionable validity. We have instead presented our findings in a narrative format with focus on the most widely reported inflammatory markers, and methodological factors.

## Results

**Fig 1:**
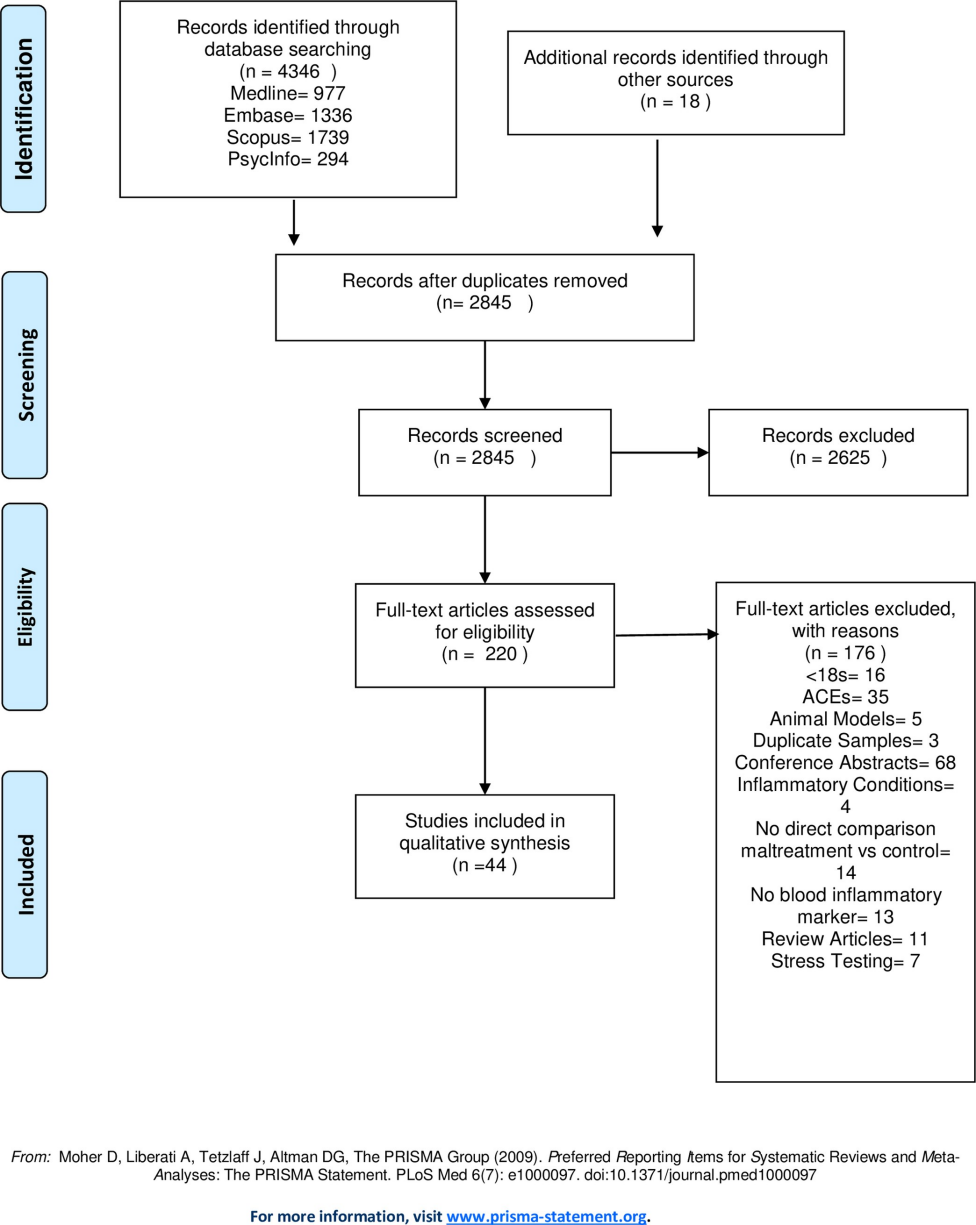
PRISMA flow diagram.

The search was conducted on 15/5/20. The PRISMA flow chart is shown in Fig 1. Details of reasons for exclusion of articles are shown in S1 Table. A total of 44 papers were included in this review. All papers were rated as moderate to high quality (CCAT scores ranged from 23–38, median- 32). There was no clear difference in the findings of studies of high or moderate quality. 30 papers reported on multiple biomarkers. The frequency with which biomarkers were reported is shown in Table 1. The most widely reported biomarkers were CRP (n = 27), IL-6 (n = 24), and TNF-a (n = 17). Details of these are discussed below. Details of other biomarkers reported are shown in S2 Table.

**Table 1:** Frequency with which biomarkers are reported in included studies.

Biomarker	Number of studies
C-reactive protein (CRP)	27
Interleukin 6 (IL-6)	24
Tumour necrosis factor alpha (TNF-a)	17
Interleukin 1b (IL-1b)	8
Interleukin 10 (IL-10)	6
Fibrinogen	4
Soluble tumour necrosis factor receptor 1 (sTNFR1)	3
Adiponectin	2
Brain derived neurotrophic factor (BDNF)	2
Interferon gamma (IFN-y)	2
Interleukin 1 receptor antagonist (IL-1RA)	2
Interleukin-4 (IL-4)	2
Resistin	2
Transforming growth factor beta (TGFB)	2
White cell count (WCC)	2
Agouiti related protein (AgRP)	1
Basic fibroblast growth factor (bFGF)	1
Betacellulin (BTC)	1
Cell-mediated immunity (EBV titre)	1
Chemokine Ligand-2 (CL-2)	1
D-Dimer	1
E-Selectin	1
Glucocorticoid induced tumour necrosis factor ligand (GITR-L)	1
Glycoprotein 130	1
Insulin-like growth factor-binding protein 2 (IGFBP2)	1
Interferon induced T-cell alpha chemoattractant (I-TAC)	1
Interleukin 2 (IL-2)	1
Interleukin 8 (IL-8)	1
Interleukin 12 (IL-12)	1
Neurotrophin-4	1
Platelet derived growth factor (PDGF)	1
Salivary alpha amylase (sAA)	1
Soluble immunoglobulin A (sIgA)	1
Soluble interleukin 6 receptor (sIL-6R)	1
Stem cell factor (SCF)	1
Thymus expressed chemokine (TECK)	1
Tumour necrosis factor related apoptosis inducing ligand-receptor 4 (TRAIL-R4)	1
Vascular endothelial growth factor (VEGF)	1
Von Wilebrand Factor (vWF)	1

### Methodological features

Over 90% (n = 41) of the 44 included studies recorded CM exposure retrospectively, with the Childhood Trauma Questionnaire (CTQ) being the most widely reported scale (n = 29; 66%). CTQ is a 28-item self-report measure of childhood trauma, which can be considered as a total score, or as subscales representing physical abuse, physical neglect, emotional abuse, emotional neglect, and sexual abuse [28]. Of papers utilising the CTQ, 13 reported only on the total score, 8 reported on subscales only, and 8 reported on both. Thirteen papers reported on the CTQ as a dichotomous variable, using a recognised cut-off point to define high versus low scores, and 16 analysed CTQ as a continuous variable. The remaining studies utilised their own measures of CM (n = 7) or other standardised scales (n = 8). After CTQ the most widely used standardised scale was the Early Trauma Inventory (ETI) (n = 3) [29]. This is a 56-item self-report scale which generates 5 variables- total number of traumas, physical trauma, emotional trauma, sexual trauma, and general traumas. General trauma includes a range of adverse exposures including parental separation, bereavement, natural disaster, and political violence. ETI studies were included if they included specific findings for subscales, allowing the specific effects of CM to be identified separately from general trauma. Most studies did not specify the timing or duration of CM in their analysis.

There was significant variation in statistical techniques used and where multivariate analysis was performed there was inconsistency as to which covariates were included. Eighty-six percent (n = 38) of the studies were conducted in high income settings (North America, Europe, Australasia, Japan), with the remainder taking place in Brazil (n = 4) and China (n = 2). Ten studies were restricted to female participants, and one was restricted to males.

### C-reactive protein

The association between CM and CRP was reported in 27 papers (full details in Table 2A and 2B). Nine reported on clinical samples and 18 on non-clinical samples.

**Table 2:** a: Papers reporting CRP: Clinical populations. b: Papers reporting CRP: Non-clinical populations.

Author	Sample details	Sample Size	Sample demographics (Age: mean (SD); Gender: No. (%))	Abuse measure	CCAT score	Main findings
Aas 2017 [35]	Participants in TOPS study of psychotic disorders in Oslo Norway. Consecutively recruited outpatients with schizophrenia and bipolar disorder and matched controls	483	Schizophrenia (n = 148) Age = 28.6 (9.3) Female = 55 (41.0%) Bipolar Disorder (n = 123) Age = 32.2 (11.7) Female = 73 (59%) Control (n = 212) Age = 30.9 (7.5) Female = 86 (41%)	CTQ- analysed as number of types of abuse. Note that neglect items were excluded.	36	In an unadjusted ANOVA CM was associated with elevated CRP in patients with psychotic disorders (Cohen’s d = 0.6, p = 0.0007) and healthy controls (Cohen’s D = 0.3, p = 0.007). When BMI was introduced to the model this association attenuated to non-significance (p>0.1) for both patients and controls.
Counotte 2019 [30]	Participants in a study of participants with psychotic disorders, ultra-high risk for psychosis (UHR), unaffected siblings, and controls, in the Netherlands.	117	Psychosis (n = 38) Age = 25.5 (23–30) Female = 6 (15.8%) UHR (n = 11) Age = 24.0 (20–29) Female = 6 (54.5%) Siblings (n = 29) Age = 25.5 (21.3–30.0) Female = 12 (41.4%) Controls (n = 39) Age = 24.0 (21.0–26.0) Female = 21 (53.8%)	CTQ- dichotomised; total and sub-components	38	CM was not associated with CRP in an unadjusted linear regression (b = -0.21; 95% CI: -0.96, 0.55; p = 0.590) or linear regression adjusted for age, sex, BMI, smoking status, cannabis use, education, and concurrent medications (b = 0.02; 95% CI: -0.81, 0.85; p = 0.960).
Fanning 2015 [36]	Community recruited participants with and without personality disorder in a larger study about the correlates of impulsive aggression in Chicago USA	134	Personality Disorder (n = 79) Age = 36.0 (7.7) Female = 38 (48.1%) Control (n = 55) Age- 31.9 (9.2) Female = 30 (54.5%)	CTQ- separated to abuse and neglect sub-components; continuous analysis.	30	In an unadjusted bivariate correlation, abuse was significantly correlated with CRP (r = 0.31; p<0.01); neglect was not significantly correlated with CRP (r = 0.16; p = NS).
Hepgul 2012 [37]	Inpatients with first episode psychosis (FEP) and healthy controls in the UK. Participants had no significant physical illnesses.	195	FEP (n = 96) Age = 27.0 (0.6) Female = 60 (57.5%) Controls (n = 99) Age = 26.3 (0.6) Female = 67 (32.3%)	CTQ- dichotomous; total and subcomponents	33	An unadjusted ANOVA compared CRP between patients with a history of CM (mean CRP = 0.8 +/-0.5) patients with no history of CM (mean CRP = 0.7 +/-0.4) and controls (mean CRP = 0.2 +/-0.1) there was no significant difference between groups (F = 2.5, df = 2,70, p = 0.089). In post-hoc testing comparison of patients exposed to sexual abuse (CRP = 1.9 +/- 2.5) patient with no exposure to sexual abuse (CRP = 0.5 +/- 0.2) and controls (CRP = 0.2 +/- 0.1) there was a significant difference between groups (F = 8.3, df = 2,189, p = 0.013).Post hoc testing showed that patients exposed to sexual abuse had greater CRP than patients not exposed to sexual abuse (p<0.001) and controls (p<0.001). Of note BMI also differed significantly between groups with greatest BMI found in patients exposed to CM.
Imai 2008 [31]	Female outpatients with PTSD and matching controls in Japan. Participants had no significant physical illnesses. Analysis of inflammation and abuse only included the PTSD group.	40 (participants with PTSD)	Age = 38.3 (10.1) Female = 100%	CTQ- total; continuous	37	No correlation of CTQ (total or subscales) with CRP in an unadjusted bivariate correlation- figures not reported in paper.
Palmos 2019 [32]	Participants retrieved from 2 studies. Controls recruited from SeLCoH study of mental and physical health in the general population in London. MDD participants recruited from the control arm of the ADD trial of metyrapone in depression. Participants had no other mental or physical illnesses.	465	MDD (n = 164) Age = 48.50 (16.14) Female = 79 (48.1%) Control (n = 301) Age = 48.50 (16.14) Female = 158 (52.5%)	CTQ total score; dichotomised	37	In a linear regression adjusted for ethnicity, smoking status, antidepressant use, study site (ADD study), plate/batch effects, current depressive episode severity, gender, age, and BMI there was no significant association of CM with CRP in controls (F = 0.002, df = 272, p = 0.960) or cases (F = 3.770, df = 141, p = 0.054). Unadjusted findings not reported.
De Punder 2018 [34]	Patients with depression were recruited from an affective disorders clinic and controls from public advertisements. Participants did not have other mental or physical illnesses. Neither patients nor controls taking psychotropic medications. Study in Berlin Germany	86	MDD and CM (n = 23) Age = 38.09 (11.36) Female = 14 (60.9%) MDD no CM (n = 23) Age = 32.61 (11.74) Female = 18 (78.2%) Control and CM (n = 21) Age = 38.09 (11.36) Female = 14 (66.7%) Control no CM (n = 21) Age = 33.9 (9.77) Female = 13 (61.9%)	ETI- Dichotomised by presence of physical or sexual abuse.	31	In an unadjusted general linear model significant effect of group effect on log CRP (F_(3,83 (_= 3.10; p = 0.031 r^2^ = 0.10). On post-hoc testing CRP levels were significantly higher in controls with CM vs controls with no CM (p-0.031) however this was no longer significant on adjustment for smoking and BMI (p = 0.052).
Quide 2019 [38]	Participants were outpatients with schizophrenia/schizoaffective disorder, bipolar disorder, and healthy controls from Austrailia. All aged 18–65 with no significant physical illnesses	209	Schizophrenia (n = 68) Age = 41.78 (11.33) Female = 29 (43%) Bipolar Disorder (n = 69) Age = 38.11 (12.31) Female = 46 (67%) Control (n = 72) Age = 36.17 (11.57) Female = 34 (47%)	CTQ- Sub-components; continuous	35	In healthy controls CRP was not association with emotional abuse (b = 0.072, b = 0.711), physical abuse (b = -0.161, p = 0.333), sexual abuse (b = 0.067, p = 0.677), emotional neglect (b = -0.079, p = 0.653) or physical neglect (b = 0.191, p = 0.343) in a multiple hierarchal linear regression adjusted for age and gender. In patients with bipolar disorder CRP was not associated emotional abuse (b = 0.176, p = 0.338), physical abuse (b = -0.136, p = 0.453), sexual abuse (b = -0.053, p-0.748), emotional neglect (b = -0.178, p = 0.348), or physical neglect (b = 0.203, p = 0.245), in a multiple hierarchal linear regression adjusted for age, sex, symptom severity, and medication use. In patients with schizophrenia CRP was significantly associated with sexual abuse (b = 0.326, p = 0.018) but not with emotional abuse (b = -0.213, p = 0.283), physical abuse (b = -0.053, p = 0.750), emotional neglect (b = -0.024, p = 0.868), or physical neglect (b = 0.009, p = 0.962) in a multiple hierarchal linear regression adjusted for age, sex, symptom severity, and medication use. Unadjusted regression coefficients not reported. Confidence intervals not reported.
Zeugmann 2013 [33]	Inpatients with MDD in Germany.	25	Age = 47.8 (15.02) Female = 17 (68%)	CTQ- subcomponents; continuous	31	In a linear regression adjusted for age and sex there was no association CRP with CM (F_5,19_ = 1.50, p0.24, adjusted R^2^ = 0.10).
Prospective Studies						
Danese 2007 [9]	Participants in longitudinal cohort study in Dunedin New Zealand. Bloods collected at visit at age 32.	892.	Age = 32 Female = 435 (48.8%)	Prospectively recorded childhood maltreatment including parental rejection and harsh discipline, and retrospective report of physical and sexual abuse.	35	In an unadjusted Cox Regression CM was associated with elevated CRP (RR = 1.80 95% CI = 1.26–2.58). This remained significant on adjustment for early life risks, stress in adulthood, and adult heath and heath behaviours (including BMI) (RR = 1.76; 95% CI = 1.23–2.51).
Nikulina 2014 [39]	Prospective cohort study of court substantiated cases of child neglect and matching controls from a midwestern city in the USA. Cases were recruited between 1967–1971.	528	Age = 41 (no SD) Female = 269 (51%)	Court substantiated cases of child neglect	29	In hierarchal linear model adjusted for age, gender, ethnicity, family and neighbourhood poverty, smoking, and BMI, neglect was not associated with CRP in total sample (OR = 1.23 95% CI- 0.83–1.84). When stratified by ethnicity neglect was significantly associated with elevated CRP in white participants only (OR = 2.18, 95% CI- 1.29–3.67); results for African-American participants not shown. African-American ethnicity was associated with elevated CRP.
Osborn 2019 [40]	Offspring of participants of a longitudinal cohort of abuse/neglect in Midwestern USA. The primary aim of this study was to assess the agreement between prospective and retrospective measures of maltreatment, and how these predict CRP.	443	Age = 23.4 (5.23) Female = 215 (48.5%)	Prospective- CPS documentation. Retrospective- LONGSCAN and Conflict Tactics Scale.	30	Prospective maltreatment was significantly associated with elevated CRP (b = 0.15, SE = 0.08, p<0.05). When analysed by abuse type this was only significant for physical abuse (b = 0.39, SE = 0.18, p<0.05). No retrospective measure was associated with CRP.
Retrospective Studies						
Anderson 2018 [41]	Mothers of participants in the ALSPAC longitudinal cohort in Bristol, UK. Mothers were invited to participate an average of 18 years after enrolment in the study.	3612	Age = 48.13 (4.35) Female = 3612 (100%)	Childhood adversity variables were extracted from questionnaire items administered earlier in course of cohort, and combined using factor analysis. The paper includes wider measures of childhood adversity, of relevance to this review are data on sexual abuse and non-sexual abuse (physical, abuse, and emotional abuse and neglect).	33	In structural equation models adjusted for age at assessment, socioeconomic stats, ethnicity, and BMI CRP was not associated with sexual abuse (b = 1.00 95%CI: 0.93, 1.08, p = 0.97) or non-sexual abuse (b = 0.98, 95%CI: 0.92, 1.04; p = 0.48).
Bertone-Johnson 2012 [50]	Participants in Nurses Health Study II. A cohort of female nurses in USA recruited in 1989. A sub-sample was invited to provide blood samples between 1996–1999.	702	Age = 48.9 (SD not reported) Female = 702 (100%)	Physical abuse- Adapted Revised Conflict Tactics Scale. Sexual abuse- Adapted Sexual Experiences Survey	33	In general linear regression CRP was not associated with child or adolescent PA in unadjusted models or models adjusted for BMI, smoking, physical activity, alcohol intake, diet, and antidepressant use. Elevated CRP was associated with adolescent SA only, in models adjusted for age, ethnicity and SES (p = 0.04) but attenuated to non-significance adjustment for BMI, smoking status, physical activity, alcohol intake, diet, and antidepressant use. Data presented as group geometric means. Regression coefficients not reported.
Boeck 2016 [42]	Cohort study of new mothers recruited in Ulm Germany. Participant had no mental or physical health problems. Bloods collected 3 months post-partum	30	Age = 31.6 (6.0) Female = 30 (100%)	CTQ- total; continuous	35	In unadjusted linear regression CRP was not associated with CRP (b = -0.12, p = 0.64).
Carpenter 2012 [43]	Sub-sample of a larger cohort study of stress and biomarkers based in Providence USA. Participants were community based with no history of mental or physical health problems.	92	Age = 30.5 (9.2) Female = 47 (51.1%)	CTQ- total and subcomponents; continuous.	35	In unadjusted bivariate correlation no correlation of CRP with total CTP (r = 0.038, p = 0.720). There was no significant correlation with any sub-component of CTQ.
Finy 2018 [53]	Pregnant woman recruited in Ohio USA. Participants were physically healthy.	214	Age = 29.38 (4.93) Female = 214 (100%)	CTQ- subscales of physical abuse, sexual abuse, and emotional abuse; continuous	35	In unadjusted bivariate correlation CTQ was positively correlated with CRP (r = 0.29, p<0.01). In structural equation models adjusted for age, gestation, pregnancy complications, and race, CM was significantly associated with CRP (beta = 0.155, p = 0.026). This effect was indirect and mediated by BMI.
Gong 2019 [44]	Study of Chinese University students exploring association of schizotypal personality traits, childhood abuse, and CRP. Participants had no physical or mental illnesses or family history of mental illness.	224	Age = 18.86 (0.86) Female = 74 (33%)	CTQ- subcomponents; continuous	31	Analyses were stratified by gender. No correlation between CTQ subscales with CRP in males or females in an unadjusted analyses.
Gouin 2012 [45]	Participants in a study of stress in older adult dementia caregivers in Canada. Participants excluded if had illnesses which affect immune system, or BMI >40.	130	CM (n = 57) Age = 62.47 (12.07) Female = 46 (80.7%) Control (n = 73) Age = 67.2 (13.74) Female = 61 (83.5%)	CTQ- total (excluding physical neglect); dichotomised	35	In a linear regression model adjusting for age, sex, ethnicity, education, BMI, marital status, sleep disturbance, and caregiving status CM was not associated with CRP (b = 0.07, SE = 0.08, p = 0.39, R2 = 0.005).
Hartwell 2013 [46]	Control group in a larger study on gender differences in stress responses in cocaine dependency in South Carolina USA. Participants were non-cocaine using controls with no mental or physical illnesses.	39	Age = 35.69 (12.0) Female = 20 (51.3%)	ETI- Number of sexual traumas, physical traumas, and emotional traumas.	36	In a linear regression model adjusted for age, sex, and smoking status, CRP was not associated with number of sexual traumas (b = 0.00, p = 1.00), physical traumas (b = -0.07, p = 0.16) or emotional traumas (b = -0.07, p = 0.04).
Kim 2019 [47]	Healthy students at a university in Texas USA.	85	Age = 21.0 (no SD) Female = 61 (71.7%)	ACEs questionnaire- specific analyses based on items on abuse and neglect	29	On unadjusted structural equation modelling abuse (b = -0.36, SE = 0.22, p = NS) and neglect (b = 0.37, SE = 0.37, p = ns) were no associated with CRP. On adjusting for age, gender, ethnicity, parental education, depressive symptoms and perceived stress, this remained the case for abuse (b = -0.43, SE = 0.24, p = NS) and neglect (b = 0.34, SE = 0.38, p = NS).
Matthews 2014 [54]	Participants in the Pittsburgh site of the SWAN cohort study of menopause and aging.	326	Age = 45.7 (2.5) Female = 326 (100%)	CTQ- total and subcomponents; dichotomised	37	In generalised estimating equations adjusted for age, race, education, smoking status, medications, cardiovascular disease, hormone therapy and depressive symptoms, SA (b = 0.03, SE = 0.01, p = 0.02), EA (0.03, SE = 0.01, p = 0.03), EN (b = 0.03, SE = 0.01, p = 0.04), PN (b = 0.05, SE = 0.02, p = 0.03) and total number of abuse types (b = 0.05, SE = 0.02, p = 0.03) were associated with higher CRP over 7 years. PA was not associated with CRP (b = 0.03, SE = 0.02, p = 0.15). All abuse measures attenuated to non-significance when BMI was introduced to the model. The effects of SA, PN, and total number of abuse type. When analysed with outcome variable of change over time in CRP, EA (b = 0.02, SE = 0.01, p = 0.005) and EN (b = 0.02, SE = 0.01, p = 0.02) were in associated with greater rises in CRP in models adjusted for BMI and other covariates.
Pinto Pereira 2019 [51]	1) 1958 British Birth Cohort (BBC): A longitudinal cohort of all born in one week in 1958. Bloods collected at visit age 45. 2) Midlife in United States (MIDUS): Cohort of adults 25–75 in USA recruited in 1994–1995. 2^nd^ wave of data collection (MIDUS-II) followed up original sample 10 years later.	British Birth Cohort = 7661 MIDUS = 1255	British Birth Cohort: Age = 45.2 (44.3–46.0) (mean and range) Female = 3828 (50%) MIDUS Age - Male = 57.9 (36–86) - Female = 56.9 (35–86) Female = 713 (56.8%)	Physical, emotional and sexual abuse; emotional neglect and physical neglect (British Birth Cohort only). Derived from study questions. All measures retrospective except from prospective record of neglect in British Birth Cohort.	25	In linear regression adjusted for age, race, gender, and other types of abuse; physical abuse was associated with increased CRP in BBC (mean percentage difference = 16.3, 95% CI = 3.01–29.7) but not MIDUS (mean percentage difference = 17.0, 95% CI = -16.4–50.3). The association of physical abuse and CRP in BBC attenuated to non-significance after adjusting for BMI (mean percentage difference = 8.41, 95% CI- -3.37, 20.2). Other forms of maltreatment were not associated with CRP in either sample.
Powers 2016 [48]	Participants were a subgroup with type 2 diabetes drawn from a larger study of risk factors for PTSD in low socioeconomic status urban African-American women in Atlanta Georgia.	40	Age = 51.88 (7.57) Female = 100%	CTQ- total; continuous	30	In an unadjusted bivariate correlation CM was not associated with CRP (r = 0.29, p = 0.068).
Rooks 2012 [52]	Male middle aged twins born between 1946–1956 from the Vietnam Era Twin Registry in USA.	482	Age = 55(3) Female = 0	ETI- Number of sexual, physical and emotional traumas.	32	On unadjusted linear regression total trauma exposure was associated with elevated CRP (b = 0.03, p = 0.03). This remained significant on adjusting for education status and income (b = 0.03, p = 0.03) but attenuated to non-significance on adjustment for BMI, blood lipids, and history of coronary heart disease (b = 0.02, p = 0.12). There was no association of CRP was any sub-type of abuse on unadjusted or fully adjusted analyses.
Schrepf 2014 [55]	Participants in MIDUS-2 biomarker project in USA.	687	Age = 52.2 (10.9) Female = 56%	CTQ- subcomponents; continuous	31	On unadjusted bivariate correlation, emotional abuse (r = 0.111, p<0.034), physical abuse (r = 0.101, p<0.304), and physical neglect (r = 0.081, p<0.034) were associated with CRP. No significant correlation with sexual abuse or emotional neglect. In structural equation modelling association of CM with CRP was mediated by BMI.
Thurston 2017 [49]	Participants in the MsHeart study of peri- and post-menopausal women aged 40–60 in Pittsburgh USA. Excluded if history of significant physical illness	286	CM (n = 132) Age = 53.68 (4.11) Female = 100%) Control (n = 163) Age = 54.28 (3.90) Female = 100%	CTQ- total and subcomponents; dichotomised	29	In unadjusted 2 sample test, no significant difference between median CRP in CM group (median = 1.28pg/ml, IQR = 0.68–4.18) versus control (median = 1.44pg/ml, IQR = 0.68–3.54), p = 0.680.

BMI- Body mass index, CCAT- Crowe Critical Appraisal Tool, CM- Child Maltreatment, CRP- C-reactive protein, CTQ- Childhood Trauma Questionnaire, EA- Emotional abuse, EN- Emotional neglect, ETI- Early Trauma Inventory, FEP- First Episode Psychosis, MDD- Major Depressive Disorder, PA- Physical abuse, PN- Physical abuse, SA- Sexual abuse, UHR- Ultra-high risk.

All papers reporting clinical samples were retrospective; eight recorded CM exposure using CTQ and one used ETI. Four studies did not find any association between CM and CRP [30–33] (data shown in Table 2A). A further two studies found initially significant associations between CM measures and CRP, which attenuated to non-significance on adjustment for BMI [34,35] (data shown in Table 2A). Significant associations between CM with CRP were reported in three studies. In a study comparing 79 participants with personality disorder and 55 healthy controls Fanning et al demonstrated a significant association between abuse and CRP, as measured retrospectively using the CTQ (r = 0.31, p<0.01), but not neglect (r = 0.16, p = NS), in a bivariate correlation which did not adjust for covariates [36]. In a sample of 96 participants with first episode psychosis and 99 healthy controls, Hepgul *et al* found a trend towards elevated CRP in patients who had experienced CM, but this only reached statistical significance when participants were grouped by exposure to sexual abuse exposure in an unadjusted ANOVA (mean CRP for patients with a history of sexual abuse, patients with no history of sexual abuse, and controls were 1.9mg/dl (SD = 0.04), 0.5mg/dl (SD = 0.2) and 0.2mg/dl (SD = 0.01) respectively, F = 8.3, df = 2,183, p = 0.013) [37]. In a study of 209 participants with schizophrenia/schizoaffective disorder, bipolar affective disorder and healthy controls, Quide et al demonstrated a significant association between sexual abuse and elevated CRP in schizophrenia patients only (b = 0.326, p = 0.018) [38]. No other associations of CRP with other abuse sub-types or in different clinical groups was demonstrated. The analysis adjusted for age, gender, disease severity, and medication use, but did not adjust for BMI.

Of 18 studies examining the association between CM and CRP in non-clinical samples, 15 were retrospective and three prospective. The three prospective studies found significant associations between CM and elevated CRP. Danese et al reported on a large prospective cohort in New Zealand that measured CM using a combination of prospective and retrospective reports [9]. They demonstrated a significant association between CM and elevated CRP (defined as >3mg/dl) which remained significant in extensively adjusted models, including adjustments for adult health behaviours and obesity (RR = 1.76, 95% CI = 1.23–2.51). They estimated that 10% of low-grade inflammation as measured by CRP may be independently attributable to CM. Nikulina et al report a US cohort exposed to court substantiated neglect and controls matched for age, sex, ethnicity, and socioeconomic status. In a model adjusting for BMI there was no significant association between neglect and elevated CRP (defined as >1mg/dl) in the total sample (OR = 1.23, 95% CI = 0.83–1.84), however the study did identify a significant interaction with race (authors’ terminology)- wherein neglect was associated with elevated CRP in white participants only (OR = 2.18, 95%CI = 1.29,3.67) [39]. Their analysis considered family poverty as a covariate in this analysis, but it did not reach significance threshold for inclusion in the final model. Osborn et al reported on the association between retrospective and prospective measures of CM with CRP [40]. They found that CRP was associated with prospective measures of CM only (b = 0.15, SE = 0.08, p<0.05). Of note their analysis adjusted for age, sex, ethnicity, parental occupation, heavy drinking, smoking, and depression but did not adjust for BMI.

Of the 15 retrospective studies in non-clinical samples, nine studies found no significant association between CM and CRP [41–49] (data shown in Table 2B). A further three found initially significant associations which attenuated to non-significance on adjustment for BMI [50–52] (data shown in Table 2B). Finy et al report a study of 214 pregnant women (of whom 51.4% were overweight or obese) in which they reported small but statistically significant associations of CTQ score with CRP (b = 0.155, p = 0.026)) [53]. Through structural equation modelling, this association was found to be indirect and mediated by elevated BMI. Similarly, Matthews et al in a middle-aged female sample, found that sexual abuse, physical neglect and total number of abuse types were associated with elevated CRP in a relationship mediated by elevated BMI. They also found that emotional abuse and neglect were independently associated with elevated CRP. This was the only paper to analyse the CRP over time, and found that emotional abuse (b = 0.02, p = 0.005), emotional neglect (b = 0.02, p = 0.02) were associated with greater rises in CRP over time [54]. Schrepf and colleagues, in a study of 687 participants in the MIDUS-II biomarker project found that CM was significantly associated with elevated CRP in adulthood in a relationship that was mediated by elevated BMI. They found that this relationship was mediated by a latent distress measure which was associated with using food as a coping mechanism. The association between BMI and CRP was stronger in females than males [55].

To summarise an association between CM and later elevation of CRP was demonstrated in three prospective studies in non-clinical samples, which adjusted for relevant covariates (however one did not adjust for BMI). Most retrospective studies (12/15) found either no association of CRP with CM or an association which attenuated to non-significance on adjustment for BMI or other obesity measures. Three studies found a significant association of CM with CRP, all of which found that this to be mediated by elevated BMI.

### Interleukin-6

The association between CM and IL-6 was reported in 24 papers, 15 of which were based on clinical samples. Details of included papers are shown in Tables 3A and 4B. All papers utilised retrospective measures of CM. Nineteen papers used CTQ to measure CM.

**Table 3:** a. Studies reporting interleukin-6: Clinical populations. b Samples reporting interleukin-6: Non-clinical populations.

Author	Sample Characteristics	Sample Size	Sample Demographics (Age: mean (SD); Gender: No. (%))	Maltreatment Measure	CCAT Score	Main Findings
Corsi-Zuelli 2020 [56]	Participants in the STREAM Cohort of patients with first episode psychosis, unaffected siblings, and healthy controls in Sao Paulo Brazil.	422	FEP (n = 114) Age = 30.8 (12.5) Female = 41 (37%) Siblings (n = 57) Age = 30.7 (10.5) Female = 39 (68.4%) Controls (n = 251) Age = 31.3 (11) Female = 122 (48.6%)	CTQ- subcomponents; dichotomised	35	In analyses adjusted for age, gender, BMI, smoking, substance use, education, and relationship status, no significant association of |l-6 with any subcomponent of abuse or neglect in any study group (p>0.05, full results not shown).
Counotte 2019 [30]	Participants in a study of participants with psychotic disorders, ultra-high risk for psychosis, unaffected siblings, and controls, in the Netherlands.	117	Psychosis (n = 38) Age = 25.5 (23–30) Female = 6 (15.8%) UHR (n = 11) Age = 24.0 (20–29) Female = 6 (54.5%) Siblings (n = 29) Age = 25.5 (21.3–30.0) Female = 12 (41.4%) Controls (n = 39) Age = 24.0 (21.0–26.0) Female = 21 (53.8%)	CTQ- total; dichotomised	38	CM was not associated with IL-6 in an unadjusted linear regression (b = -1.42; 95% CI: -3.03, 0.20; p = 0.084) or linear regression adjusted for age, sex, BMI, smoking status, cannabis use, education, and concurrent medications (b = -1.14; 95% CI: -2.99, 0.71; p = 0.224).
Dennison 2012 [60]	Patients with schizophrenia in Cork Ireland recruited from outpatient and inpatient settings. Controls recruited from local university.	80	Schizophrenia (n = 40) Age = 38.33 (1.7) Female = 16 (40%) Controls (n = 40) Age = 36.2 (1.76) Female = 27 (67.5%)	CTQ- total; dichotomised	31	Higher levels of IL-6 in patients with schizophrenia who reported exposure to CM (mean = 2.149, SD = 0.318) compared to patients with schizophrenia who did not report CM (mean = 1.048, SD = 0.172) and healthy controls (mean = 1.092, SD = 0.296), in an unadjusted ANOVA (F = 4.258, df = 143, p<0.05).
Fanning 2015 [36]	Community recruited participants with and without personality disorder in a larger study about the correlates of impulsive aggression in Chicago USA	134	Personality Disorder (n = 79) Age = 36.0 (7.7) Female = 38 (48.1%) Control (n = 55) Age- 31.9 (9.2) Female = 30 (54.5%)	CTQ- separated to abuse and neglect sub-components; continuous analysis.	30	In an unadjusted bivariate correlation there was no significant correlation of IL-6 with abuse (r = 0.13, p = NS) or neglect (r = 0.12, p = NS)
Grosse 2016 [61]	Participants in the MOODINFLAME study of inflammatory biomarkers in MDD. Participants were inpatients with MDD in hospitals in Munster Germany. Participants were free from other mental illness or significant physical illnesses. Controls were recruited from the community.	394	MDD (n = 214) Age = 41 (12) Female = 120 (56%) Control (n = 180) Age = 36 (12) Female = 114 (63%)	CTQ- total and subcomponents; dichotomised	30	In linear regression no association of CM with IL-6 in a model adjusting for age, gender, waist hip ratio, and smoking in depressed participants (b = 0.033, p = 0.649) or controls (b = 0.025, p = 0.751). In a post hoc analysis SA was associated with elevated IL-6 in participants with MDD and CM.
Imai 2008 [31]	Female outpatients with PTSD and matching controls in Japan. Participants had no significant physical illnesses. Analysis of inflammation and abuse only included the PTSD group.	40 (participants with PTSD)	Age = 38.3 (10.1) Female = 100%	CTQ- total; continuous	37	No correlation of CTQ (total or subscales) with any inflammatory marker in unadjusted analysis- figures not reported in paper.
Lu 2013 [57]	Study of outpatients with MDD (with and without and history of childhood maltreatment) and controls recruited from Changsha China. Participants had no other mental or physical illnesses.	65	MDD and CM (n = 22) Age = 30.2 (8.53) Female = 14 (63.6%) MDD no CM (n = 21) Age = 30.1 (6.81) Female = 11 (52.3%) Control (n = 22) Age = 27.7 (4.78) Female = 11 (50%)	CTQ- total; dichotomised	. 24	No correlation of CTQ with any cytokine in MDD and CM group in an unadjusted analysis. Data not shown.
Pedrotti Moreira 2018 [62]	Cross-sectional study of participants with MDD in Pelotas Brazil. Participants had no other mental or physical illness.	166	Age = 25.87 (5.25) Female = 124 (74.7%)	CTQ- total; continuous	34	In depressed participants CM was associated with elevated IL-6. This remained significant on linear regression adjusting for schooling and smoking status (b = 0.156, CI = 0.10–0.225, p = 0.050).
Muller 2019 [63]	Inpatient and outpatients with MDD and matching controls (from local university) recruited in Munich Germany. No other mental or physical illnesses.	88	MDD (n = 27) Age = 39.2 (12) Female = 17 (39%) Control (n = 27) Age = 38.9 (11.8) Female = 17 (39%)	CTQ- subcomponents; continuous	35	In total sample IL-6 was positively correlated with Sexual abuse (tau = 0.177, p = 0.047) and physical neglect (tau = 0.240, p = 0.006). sub-components only (data for other subscales not shown). In the control group no sub-components correlated with IL-6. (data not shown) In MDD group physical neglect correlated with IL-6 only (tau = 0.316, p = 0.013). Analyses were not adjusted for covariates.
Munjiza 2018 [64]	Inpatients with depression and matching controls in Belgrade Serbia.	117	MDD (n = 64) Age = 45.98 (10.31) Female = 51 (80.0%) Control (n = 53) Age = 46.04 (10.15) Female = 43 (81.1%)	CTQ- total and subcomponents; continuous	30	In the patient group only, IL-6 was positively correlated with total CTQ (r = 0.379, p<0.01)and PN (r = 0.323, p<0.01), EA (r = 0.382, p<0.01), and PA (r = 0.320, p<0.01) sub-components. IL-6 was not correlated with any CTQ component in controls. Analyses were not adjusted for covariates.
Palmos 2019 [32]	Participants retrieved from 2 studies. Controls recruited from SeLCoH study of mental and physical health in the general population in London. MDD participants recruited from the control arm of the ADD trial of metyrapone in depression. Participants had no other mental or physical illnesses.	465	MDD (n = 164) Age = 48.50 (16.14) Female = 79 (48.1%) Control (n = 301) Age = 48.50 (16.14) Female = 158 (52.5%)	CTQ- total; dichotomised	37	In a linear regression adjusted for ethnicity, smoking status, antidepressant use, study site (ADD study), plate/batch effects, current depressive episode severity, gender, age, and BMI there was no significant association of CM with IL-6 in controls (F = 0.030, df = 265, p = 0.864) or cases (F = 1.693, df = 138, p = 0.195). Unadjusted findings not reported.
Porcu 2018 [58]	Women aged 18–65 in Londrina Brazil. Participants were patient with unipolar or bipolar depression recruited from outpatient clinics, non-depressed smokers were recruited from smoking cessation clinics and non-smoking controls from hospital staff. Participants excluded if had other mental or physical illness	159	Depressed Smokers (n = 24) Age = 46.07(10.58) Female = 100% Depressed Never-Smokers (n = 38) Age = 41.08 (13.61) Female = 100% Control Smokers (n = 24) Age = 43.54 (11.85) Female = 100% Control Never-smokers (n = 28) Age = 39.21 (13.19) Female = 100%	CTQ- subcomponents; continuous	23	No association of CM with IL-6 in unadjusted analysis (data not shown).
De Punder 2018 [34]	Patients with depression were recruited from an affective disorders clinic and controls from public advertisements. Participants did not have other mental or physical illnesses. Neither patients or controls taking psychotropic medications. Study in Berlin Germany	86	MDD and CM (n = 23) Age = 38.09 (11.36) Female = 14 (60.1%) MDD no CM (n = 23) Age = 32.61 (11.74) Female = 18 (85.7%) Control and CM (n = 21) Age = 34.05 (10.53)) Female = 14 (66.7%) Control no CM (n = 21) Age = 33.9 (9.77) Female = 13 (61.2%)	ETI- Dichotomised by presence of physical or sexual abuse.	31	In an unadjusted general linear model significant effect of group effect on log IL-6 (F_(3,83 (_= 3.32, p = 0.024 r^2^ = 0.11). On post-hoc testing IL-6 levels were significantly MDD patients exposed to CM vs controls with no CM (p = 0.018) and this remained significant after controlling for BMI and smoking (p = 0.044).
Quide 2019 [38]	Participants were outpatients with schizophrenia/schizoaffective disorder, bipolar disorder, and healthy controls from Austrailia. All aged 18–65 with no significant physical illnesses	209	Schizophrenia (n = 68) Age = 41.78 (11.33) Female = 29 (42.6%) Bipolar Disorder (n = 69) Age = 38.11 (12.31) Female = 46 (66.7%) Control (n = 72) Age = 36.17 (11.57) Female = 34 (47.2%)	CTQ- Subcomponents; continuous	35	In controls IL-6 was not associated with emotional abuse (b = 0.070, p = 0.724), physical abuse (b = -0.025, p = 0.881), sexual abuse (b = -0.121, p = 0.451), emotional neglect (b = -0.217, p = 0.220), or physical neglect (b = 0.191, p0.343) in a multiple hierarchal linear regression adjusted for age and gender. In patients with bipolar disorder IL-6 was not associated with emotional abuse (b = 0.084, p = 0.620), physical abuse (b = 0.228, p = 0.186), sexual abuse (b = -0.319, p = 0.033), emotional neglect (b = -0.233, p = 0.186), or physical neglect (b = 0.154, p = 0.306) in a multiple hierarchal linear regression adjusting for age, sex, symptom severity, and medication use. In patients with schizophrenia IL-6 was not associated with emotional abuse (b = 0.099, p = 0.643), physical abuse (b = -0.081, p = 0.653), sexual abuse (b = 0.184, p = 0.203), emotional neglect (b = -0.123, p = 0.451), or physical neglect (b = -0.115, p = 0.556) in a multiple hierarchal linear regression adjusting for age, sex, symptom severity and medication use. Unadjusted regression coefficients not reported. Confidence intervals not reported.
Smith 2011 [59]	Participants in a larger study of influence of genetics and environmental responses to stress in African Americans in Atlanta Georgia. Participants grouped by presence of PTSD and early maltreatment Also describe “extended sample” of 177- unclear origin	110	Sample demographics not reported. States that groups were matched for age and gender.	CTQ- Total; dichotomised	27	Maltreatment was not associated with IL-6 in an analysis adjusted for age, gender, socioeconomic status, mental health outcomes, but not BMI. Data not shown.
Bertone-Johnson 2012 [50]	Participants in Nurses Health Study II. A cohort of female nurses in USA recruited in 1989. A sub-sample was invited to provide blood samples between 1996–1999.	702.	Age = 48.9 (SD not reported) Female = 100%	Physical abuse- Adapted Revised Conflict Tactics Scale. Sexual abuse- Adapted Sexual Experiences Survey	33	IL-6 was associated with adolescent PA and SA only but both became non-significant in models adjusting for BMI. Data presented as group geometric means. Regression coefficients not reported.
Boeck 2016 [42]	Cohort study of new mothers recruited in Ulm Germany. Participant had no mental or physical health problems. Bloods collected 3 months post-partum	30	Age = 31.6 (6.0) Female = 100%	CTQ- total; continuous	35	In unadjusted linear regression CM was not associated with IL-6 (b = -0.30, p = 0.15)
Davis 2019 [65]	Participants in a study of healthy aging in Arizona USA.	770.	Age = 53.5 (7.24) Female = 423 (55%)	CTQ- 10 item short version excluding neglect items; continuous	34	CM was associated with elevated IL-6 (standardised path coefficient = 0.142, SE = 0.041, p<0.001) in a model adjusted for age, gender, ethnicity, education, smoking, alcohol use, sleep disturbance, and physical activity levels but not BMI.
Finy 2018 [53]	Pregnant woman recruited in Ohio USA. Participants were physically healthy.	214	Age = 29.38 (4.93) Female = 100%	CTQ- subscales of physical abuse, sexual abuse, and emotional abuse; continuous	35	In unadjusted bivariate correlation CTQ was positively correlated with IL-6 (r = 0.30, p<0.01). In structural equation models adjusted for age, gestation, pregnancy complications, and race, CM was significantly associated with IL-6 (data not shown).
Gouin 2012 [45]	Participants in a study of stress in older adult dementia caregivers in Canada. Participants excluded if had illnesses which affect immune system, or BMI >40.	130	CM (n = 57) Age = 62.47 (12.07) Female = 46 (80.7%) Control (n = 73) Age = 67.2 (13.74) Female = 61 (83.5%)	CTQ- total (excluding physical neglect); dichotomised	35	In a linear regression model adjusting for age, sex, ethnicity, education, BMI, marital status, sleep disturbance, and caregiving status, CM was significantly associated with elevated IL-6 (b = 0.09, SE = 0.03, p = 0.01, R^2^ = 0.055).
Hartwell 2013 [46]	Control group in a larger study on gender differences in stress responses in cocaine dependency in South Carolina USA. Participants were non-cocaine using controls with no mental or physical illnesses.	39	Age = 35.69 (12.0) Female = 20 (51.3%)	ETI- Number of sexual, physical, and emotional traumas.	36	In a linear regression model adjusted for age, sex, and smoking, total number of traumas was associated with elevated IL-6 (F_1,30_ = 4.05, p = 0.05). When analysed by subscales this was driven by an association of IL-6 with general trauma. IL-6 was not significantly associated with number of sexual traumas (b = -0.04, p = 0.41), physical traumas (b = 0.01, p = 0.88), or emotional traumas (b = -0.02, p = 0.65).
Kiecolt-Glaser 2011 [66]	Participants in a study of caregiving stress in older adults including caregivers for a spouse or parent with dementia and matching controls, in Ohio USA. Participants had no immune-related health problems.	132	Age = 69.7 (10.14) Female = 95 (72%)	CTQ total (excluding physical neglect); dichotomised	36	Abuse was associated with increased IL-6 (F(1,126) = 9.51, p = 0.003). in a model adjusted for age, sex, BMI, marital status, and caregiver status.
Pinto Pereira 2019 [51]	1) 1958 British Birth Cohort: A longitudinal cohort of all born in one week in 1958. Bloods collected at visit age 45. 2) Midlife in United States (MIDUS): Cohort of adults 25–75 in USA recruited in 1994–1995. 2^nd^ wave of data collection (MIDUS-II) followed up original sample 10 years later.	British Birth Cohort = 7661 MIDUS = 1255	British Birth Cohort:Age = 45.2 (44.3–46.0) (mean and range)Female = 3828 (50%)MIDUS Age • Male = 57.9 (36–86) • Female = 56.9 (35–86) Female = 713 (56.8%)	Physical, emotional and sexual abuse; emotional neglect and physical neglect (British Birth Cohort only). Derived from study questions. All measures retrospective except from prospective record of neglect in British Birth Cohort	25	No form of maltreatment was associated with differences in IL-6 in models adjusted for age, gender, ethnicity, socioeconomic status, and BMI or waist hip ratio.
Rooks 2012 [52]	Male middle aged twins born between 1946–1956 from the Vietnam Era Twin Registry in USA.	482	Age = 55(3) Female = 0	ETI- number of sexual, physical, and emotional traumas	32	In linear regression analysis total trauma exposure was not associated with IL-6 in unadjusted analysis (b = 0.01, p = 0.12) or in models adjusted for income, education, cardiovascular risk factors (including BMI). There was no association of IL-6 with any trauma subscale on unadjusted or adjusted analyses.
Thurston 2017 [49]	Participants in the MsHeart study of peri- and post-menopausal women aged 40–60 in Pitsburgh USA. Excluded if history of signficiant physical illness	286	CM (n = 132) Age = 53.68 (4.11) Female = 100% Control (n = 163): Age = 54.28 (3.90) Female = 100%	CTQ- total and subcomponents; dichotomised	29	In unadjusted 2 sample test no significant difference between CM group (median = 1.56pg/ml IQR = 1.04–2.31) versus controls (median = 1.35pg/ml, IQR = 0.94–2.23), p = 0.189.

BMI- Body mass index, CCAT- Crowe Critical Appraisal Tool, CM- Child Maltreatment, CTQ- Childhood Trauma Questionnaire, EA- Emotional abuse, EN- Emotional neglect, ETI- Early Trauma Inventory, IL-6- Interleukin-6, MDD- Major depressive disorder, PA- Physical abuse, PN- Physical abuse, SA- Sexual abuse.

**Table 4:** a- Studies reporting TNF-a: Clinical populations. b- Studies reporting TNF-a: Non-clinical populations.

Author	Sample Characteristics	Sample Size	Sample Demographics	Maltreatment Measure	CCAT Score	Main Findings
Corsi-Zuelli 2020 [56]	Participants in the STREAM Cohort of patients with first episode psychosis, unaffected siblings, and healthy controls in Sao Paulo Brazil.	422	FEP (n = 114) Age = 30.8 (12.5) Female = 41 (37%) Siblings (n = 57) Age = 30.7 (10.5) Female = 39 (68.4%) Controls (n = 251) Age = 31.3 (11) Female = 129 (48.6%)	CTQ- subcomponents; dichotomised	35	In analyses adjusted for age, gender, BMI, smoking, substance use, education, and relationship status, no significant association of TNF-a with any subcomponent of abuse or neglect in any study group (p>0.05, full results not shown)
Counotte 2019 [30]	Participants in a study of participants with psychotic disorders, ultra-high risk for psychosis, unaffected siblings, and controls, in the Netherlands.	117	Psychosis (n = 38) Age = 25.5 (23–30) Female = 6 (15.8%) UHR (n = 11) Age = 24.0 (20–29) Female = 6 (54.5%) Siblings (n = 29) Age = 25.5 (21.3–30.0) Female = 12 (41.4%) Controls (n = 39) Age = 24.0 (21.0–26.0) Female = 21 (53.8%)	CTQ- dichotomised; total and subcomponents	38	CM was not associated with TNF-a in an unadjusted linear regression (b = -0.48; 95% CI: -1.37, 0.42; p = 0.21) or linear regression adjusted for age, sex, BMI, smoking status, cannabis use, education, and concurrent medications (b = -0.75; 95% CI: -1.84, 0.35; p = 0.179).
Dennison 2012 [60]	Patients with schizophrenia in Cork Ireland recruited from outpatient and inpatient settings. Controls recruited from local university.	80	Schizophrenia (n = 40) Age = 38.33 (1.7) Female = 16 (40%) Controls (n = 40) Age = 36.2 (1.76) Female = 27 (67.5%)	CTQ- total; dichotomised	31	Higher levels of TNF-a in patients with schizophrenia who reported exposure to CM (mean = 8.248, SD = 0.601) compared to patients with schizophrenia who did not report CM (mean = 6.088, SD = 0.465) and healthy controls (mean = 3.614, SD = 0.331), in an unadjusted ANOVA (F = 11.41, df = 143, p<0.001).
Grosse 2016 [61]	Participants in the MOODINFLAME study of inflammatory biomarkers in MDD. Participants were inpatients with MDD in hosptials in Munster Germany. Participants were free from other mental illness or significant physical illnesses. Controls were recruited from the community.	394	MDD (n = 214) Age = 41 (12) Female = 120 (56%) Control (180) Age = 36 (12) Female = 114 (63%)	CTQ- total and subcomponents; dichotomised	30	In linear regression no association of CM with TNF-a in a model adjusting for age, gender, waist hip ratio, and smoking in depressed participants (b = -0.035, p = 0.638) or controls (b = 0.-0.059, p = 0.447). In a post hoc analysis SA was associated with elevated TNF-a in participants with MDD and CM.
Imai 2008 [31]	Female outpatients with PTSD and matching controls in Japan. Participants had no significant physical illnesses. Analysis of inflammation and abuse only included the PTSD group.	40 (participants with PTSD)	Age = 38.3 (10.1) Female = 100%	CTQ- total; continuous	. 37	No correlation of CTQ (total or subscales) with any inflammatory marker in unadjusted analysis- figures not reported in paper.
Kraav 2019 [67]	Sub-sample of the NeuroDep study of MDD in Kuopio Finland. All participants had MDD and had no other mental or physical illnesses.	78	CM (n = 24) Age = 41.63 (9.7) Female = 12 (50%) Control (n = 54) Age = 37.24 (12.5) Female = 30 (55.6)	ACEs questionnaire. Due to low sample size only included physical abuse item.	32	TNF-a did not differ between groups in an unadjusted analysis. PA = 11.29 (95%CI = 7.97–13.01) vs control = 11.80 (8.71–19.28); p = 0.49
Lu 2013 [57]	Study of outpatients with MDD (with and without and history of childhood maltreatment) and controls recruited from Changsha China. Participants had no other mental or physical illnesses.	65	MDD and CM (n = 22) Age = 30.2 (8.53) Female = 14 (63.6%) MDD no CM (n = 21) Age = 30.1 (6.81) Female = 11 (52.3%) Control (n = 22) Age = 27.7 (4.78) Female = 11 (50%)	CTQ- total; dichotomised	24	No correlation of CTQ with TNF-a in any group, in an unadjusted analysis. Data not shown.
Pedrotti Moreira 2018 [62]	Cross-sectional study of participants with MDD in Pelotas Brazil. Participants had no other mental or physical illness.	166	Age = 25.87 (5.25) Female = 124 (74.7%)	CTQ- total; continuous	34	There was no association of abuse and TNF-a in model adjusting for age, smoking status, and schooling.
Palmos 2019 [32]	Participants retrieved from 2 studies. Controls recruited from SeLCoH study of mental and physical health in the general population in London. MDD participants recruited from the control arm of the ADD trial of metyrapone in depression. Participants had no other mental or physical illnesses.	465	MDD (n = 164) Age = 48.50 (16.14) Female = 79 (48.1%) Control (n = 301) Age = 48.50 (16.14) Female = 158 (52.5%)	CTQ total score; dichotomised	37	In a linear regression adjusted for ethnicity, smoking status, antidepressant use, study site (ADD study), plate/batch effects, current depressive episode severity, gender, age, and BMI there was no significant association of CM with TNF-a in controls (F = 0.464, df = 282, p = 0.496) or cases (F = 0.073, df = 139, p = 0.787). Unadjusted findings not reported.
Quide 2019 [38]	Participants were outpatients with schizophrenia/schizoaffective disorder, bipolar disorder, and healthy controls from Austrailia. All aged 18–65 with no significant physical illnesses	209	Schizophrenia (n = 68) Age = 41.78 (11.33) Female = 29 (42.6%) Bipolar Disorder (n = 69) Age = 38.11 (12.31) Female = 46 (66.7%) Control (n = 72) Age = 36.17 (11.57) Female = 34 (47.2%)	CTQ- Sub-components; continuous	35	In controls TNF-a was not associated with emotional abuse (b = -0.020, p = 0.912), physical abuse (b = -0.284, p = 0.073), sexual abuse (b = 0.102, p = 0.494), emotional neglect (b = 0.091, p = 0.577), or physical neglect (b = 0.094, p = 0.617) in a multiple hierarchal linear regression adjusted for age and gender. In patients with bipolar disorder TNF-a was not associated with emotional abuse (b = -0.257, p = 0.104), physical abuse (b = 0.167, p = 0.297), sexual abuse (b = -0.161, p = 0.242), emotional neglect (b = 0.559, p = 0.955), or physical neglect (b = -0.008, p = 0.955) in a multiple hierarchal linear regression adjusting for age, sex, symptom severity, and medication use. In patients with schizophrenia TNF-a was not associated with emotional abuse (b = -0.056, p = 0.794), physical abuse (b = -0.114, p = 0.526), sexual abuse (b = 0.070, p = 0.627), emotional neglect (b = 0.040, p = 0.806), or physical neglect (b = 0.035, p = 0.861) in a multiple hierarchal linear regression adjusting for age, sex, symptom severity and medication use. Unadjusted regression coefficients not reported. Confidence intervals not reported.
Smith 2011 [59]	Participants in a larger study of influence of genetics and environmental responses to stress in African Americans in Atlanta Georgia. Participants grouped by presence of PTSD and early maltreatment	110	Sample demographics not reported. States that groups were matched for age and gender.	CTQ- Total; dichotomised	27	CM associated with TNF-a (t = 2.42, p = 0.017) but no other inflammatory markers in an analysis adjusted for age, gender, socioeconomic status, mental health outcomes, but not BMI.
Toft 2018 [68]	Patients with MDD admitted to a psychiatric hospital in Norway.	128	Mild MDD (n = 28) Age = 39.4 (10.8) Female = 20 (71.4%) Moderate MDD (n = 42) Age = 44.2 (12.0) Female = 28 (66.7%) Severe MDD (n = 58) Age = 41.3 (11.7) Female = 44 (75.9%)	No formal measure. Asked about child maltreatment on admission interview and grouped as trauma present or absent.	27	In an unadjusted two sample test TNF-a was not significantly different between CM (median = 0.920pg/ml; IQR = 0.046–3.069) vs control (median = 0.090pg/ml (IQR = 0.046–1.060), p = 0.060.
Zeugmann 2013 [33]	Inpatients with MDD in Germany.	25	Age = 47.8 (15.02) Female = 17 (68%)	CTQ- subcomponents; continuous	31	In a linear regression adjusted for age and sex TNF-a was not associated with CM (F_5,19_ = 1.09, p = 0.40, adjusted R^2^ = 0.02).
Boeck 2016 [42]	Cohort study of new mothers recruited in Ulm Germany. Participant had no mental or physical health problems. Bloods collected 3 months post-partum	30	Age = 31.6 (6.0) Female = 100%	CTQ- total; continuous	35	In unadjusted linear regression C< was not associated with TNF-a (b = 0.32, p = 0.11).
Gouin 2012 [45]	Participants in a study of stress in older adult dementia caregivers in Canada. Participants excluded if had illnesses which affect immune system, or BMI >40.	130 Abused = 57 Non-Abused = 73	CM (n = 57) Age = 62.47 (12.07) Female = 46 (80.7%) Control (n = 73) Age = 67.2 (13.74) Female = 61 (83.5%)	CTQ- total (excluding physical neglect); dichotomised	35	In a linear regression model adjusted for age, sex, ethnicity, education, BMI, marital status, sleep disturbance and caregiving status, CM was not associated with TNF-a (b = 0.06, SE = 0.03, p = 0.07, R^2^ = 0.3).
Hartwell 2013 [46]	Control group in a larger study on gender differences in stress responses in cocaine dependency in South Carolina USA. Participants were non-cocaine using controls with no mental or physical illnesses.	39	Age = 35.69 (12.0) Female = 20 (51.3%)	ETI- Number of sexual, physical, and emotional traumas.	36	In a linear regression model adjusted for age, sex, and smoking status, number of traumas was associated with elevated TNF-a (F_1,25_ = 7.86, p = 0.01). On analysis of subscales, none were significantly associated with TNF-a. There was no association of TNF-a with sexual trauma (b = 0.11, p = 0.14), physical trauma (b = 0.14, p = 0.24) or emotional trauma (b = 0.06, p = 0.36).
Kiecolt-Glaser 2011 [66]	Participants in a study of caregiving stress in older adults including caregivers for a spouse or parent with dementia and matching controls, in Ohio USA. Participants had no immune-related health problems.	132	Age = 69.7 (10.14) Female = 95 (72%)	CTQ Total (excludes physical neglect); dichotomised	36	CM was not associated with TNF-a (F(1,124) = 1.75), in a model adjusted for age, sex, BMI, marital status, and caregiver status.

BMI- Body mass index, CCAT- Crowe Critical Appraisal Tool, CM- Child Maltreatment, CTQ- Childhood Trauma Questionnaire, EA- Emotional abuse, EN- Emotional neglect, ETI- Early Trauma Inventory, MDD- Major depressive disorder, PA- Physical abuse, PN- Physical abuse, TNF-a- Tumour Necrosis Factor Alpha, SA- Sexual abuse.

Of the clinical samples, nine papers did not identify a significant association between CM and IL-6 [30–32,36,38,56–59] (data shown in Table 3A). Dennison et al reported higher levels of IL-6 in patients with schizophrenia who reported exposure to CM compared to patients with schizophrenia who did not report maltreatment and healthy controls (mean IL-6: 2.14pg/ml (SD = 0.318), 1.048pg/ml (SD = 0.172), 1.092pg/ml (SD = 0.296) respectively; F = 4,258, df = 143, p<0.05), in an ANOVA that did not adjust for covariates [60]. Grosse et al reported on 394 patients and controls in the MOODINFLAME study of inflammatory markers in Major Depressive Disorder (MDD) [61]. There was no association between CM and IL-6 in the total sample, nor in the MDD or control groups. In an analysis limited to MDD patients exposed to CM, sexual abuse was associated with elevated IL-6 in an analysis that adjusted for age, gender, smoking, and waist-hip ratio. Pedrotti Moreira et al reported on a cross-sectional study of MDD and healthy control participants in Brazil [62]. They identified a significant association between CM and higher IL-6 in participants with MDD only (b = 0.156, 95%CI = 0.10–0.225, p = 0.05), in an analysis which adjusted for education and smoking status but not BMI. Muller et al examined the correlation between CTQ scores and inflammatory markers in a sample consisting of patients with MDD and healthy controls [63]. They found a small but significant correlation between sexual abuse (tau = 0.177, p = 0.047) and physical neglect (tau = 0.240, p = 0.006) with IL-6 in an unadjusted analysis. Munjiza reported that IL-6 was positively correlated with total CTQ (r = 0.379, p<0.01), physical neglect (r = 0.323, p<0.01), emotional abuse (r = 0.382, p<0.01), and physical abuse (r = 0.320, p<0.01) in an unadjusted analysis limited to participants with MDD only [64]. De Punder et al reported on a sample of patients with MDD and healthy controls [34]. They grouped participants by presence of MDD and exposure to CM and identified a significant between group difference in an analysis which adjusted for BMI and smoking (F_3,83_ = 3.32, p = 0.024). On post-hoc testing the only significant difference was between the MDD and CM group vs healthy control and no CM, thus this analysis does not clearly distinguish the effects of MDD from CM.

Ten studies reported on non-clinical samples. Three of these found no association between CM and IL-6 [42,51,52]; a further three studies found associations of CM with elevated IL-6 which attenuated to non-significance after adjustment for BMI [49,50,53] (data shown in Table 3B). Davis et al, in a study of healthy middle aged adults in the USA, found that CM was significantly associated with elevated IL-6 (standardised path coefficient = 0.142, SE = 0.041, p<0.001) in a model that adjusted for age, gender, ethnicity, and health behaviours, but not BMI [65]. Gouin et al, in a study of care-giver stress in older adults found a significant association between CM and IL-6 (b = 0.09, SE = 0.03, p = 0.01) in a model which adjusted for age, sex, ethnicity, education, BMI and social factors [45]. Hartwell *et al* reported a significant association between the total number of traumas as measured by the ETI and elevated IL-6 (F_1,30_ = 4.05, p = 0.05) in a model which adjusted for age, sex, and smoking status but not BMI [46]. However when analysed by trauma sub-types this relationship was only significant for general trauma (which includes wider adversity like bullying) and was not significantly association with physical abuse, sexual abuse or emotional abuse. In another study of care-giver stress in older adults, Kiecolt-Glaser identified a significant association between CM and elevated IL-6 (F_1,126_ = 9.51, p = 0.003) in a model adjusted for age, sex, BMI and social factors [66].

In summary most studies did not find a significant association between CM and elevated IL-6. Studies reporting positive findings tended not to adjust for BMI and in some papers positive associations were limited to sub-groups. Notably, two studies that adjusted appropriately for covariates found significant associations between CM and elevated IL-6 in older adults.

### Tumour necrosis factor-alpha

The association between CM with TNF-a was reported in 17 papers, 13 of which were in clinical samples. All studies were retrospective and 14 used the CTQ to measure CM. Details of included papers are shown in Table 4A and 4B.

Eleven papers, all reporting clinical samples, did not identify a significant association between CM and TNF-a [30–33,38,56,57,61,62,67,68] (data shown in Table 4A). Dennison et al reported elevated levels of TNF-a in participants with schizophrenia and a history of CM compared to participants with schizophrenia with no history of CM, and controls (mean TNF-a 8.248pg/ml (SD = 0.601), 6.088pg/ml (SD = 0.465), and 3.614pg/ml (SD = 0.331) respectively, F = 11.41, df = 143, p<0.001), in an ANOVA which did not adjust for covariates [60]. Smith et al reported that TNF-a was associated with elevated TNF-a in a sample of 110 African-Americans with and without PTSD, in an analysis which adjusted for age, gender, education, substance use, mental health factors, but not BMI [59].

In non-clinical samples, two studies did not find a significant association between CM and TNF-a [45,66] (data shown in Table 4B). Hartwell et al in a study of 39 healthy adults in the USA, reported a significant association between the number of traumas on the ETI and elevated TNF-a in an analysis which adjusted for age, sex, and smoking status but not BMI [46]. This association was not statistically significant for any subscale of the ETI (including general trauma), and there was no statistically significant association of TNF-a with physical abuse, sexual abuse, or emotional abuse.

To summarise most studies did not find a significant association between CM and elevated TNF-a, and none of the studies reporting significant associations had adjusted for BMI.

### Findings relating to other biomarkers

Details of papers relating to additional biomarkers and their main findings are shown in S2 Table. The most widely reported biomarkers were IL-1b (n = 8), IL-10 (n = 5), and fibrinogen (n = 4). Most papers did not demonstrate significant associations of CM with the biomarkers studied. The most consistent association was of CM with fibrinogen which was demonstrated in three papers [9,33,51].

## Discussion

This systematic review examining the association between CM and markers of systemic inflammation has identified significant variation in the conduct and statistical analysis of studies in this area to the extent that quantitative synthesis of the findings would be invalid. Of note, there was wide variation in how CM exposure was recorded and analysed; for example, as a dichotomous versus a continuous variable; as an overall construct versus its subcomponents. Furthermore, the method of analysis varied widely, including simple between group comparisons, bivariate correlations, linear regression, and more complex modelling. Of note, in analyses where adjustment for covariates was possible there was no consistency as to which variables were included. Unsurprisingly, in this context, the findings of studies in this field are inconsistent: the majority of retrospective studies showed no association between CM and inflammatory markers, a number of unadjusted analyses showed statistically significant associations, and a smaller number of fully adjusted analyses showed statistically significant associations but with generally small effect sizes. The variation in conduct and analysis of studies makes it challenging to integrate these disparate findings into a cohesive whole.

This review highlights several limitations in the existing literature. Only three studies (less than 10%) included prospective measures of CM [9,39,40], and these studies only related to CRP. All three of these studies found a significant association between CM and elevated CRP later in life. Baldwin et al have highlighted that retrospective and prospective measures of CM tend to capture different groups of individuals and are not clearly measuring the same construct [69]. This is further supported by the findings of Osborn and colleagues who found that prospective but not retrospective measures of CM were associated with elevated CRP [40]. Despite this small prospective evidence base, and its narrow focus on CRP, the existence of these appropriately adjusted prospective studies demonstrating an association between CM and later increases in CRP suggests that further examination of the links between CM and inflammation is still warranted, but only if studies have sufficient methodological rigour.

In the research base as a whole, studies were inconsistent in their construct of CM: an overall “CM” construct versus sub-types of maltreatment; as a dichotomous variable treating CM as present or absent, or as a continuous measure of the severity of CM. Statistical properties of the way the construct of CM is presented and analysed may contribute to important differences in results (e.g. analyses of continuous measures have more statistical power than dichotomous variables). Studies were also inconsistent in their reporting and analysis of sub-types of abuse. Studies describing results for individual sub-types of CM have reported different effects for different types of maltreatment (most commonly stronger associations of sexual abuse with inflammation) [35,37,38,50,61]. An approach based on individual sub-types of maltreatment may, however, neglect the inherent complexity and clustering of adversities. For example rather than a specific effect of child sexual abuse as opposed to other maltreatment, the associations found between sexual abuse and inflammation may be more reflective of sexual abuse exposure indexing an overall greater severity of maltreatment exposure and a clustering of multiple adversities [70,71]. There were limited data on the timing and duration of CM which limits the ability to draw conclusions about sensitive periods in the development of the immune system. Overall, the inconsistencies in measurement of CM could be masking potentially important findings, especially regarding mechanisms.

The conceptualisation and measurement of CM and ACEs more broadly is an area of ongoing debate with relevance to study methodology in this area. Total scores based on the number of forms of CM or ACEs a person has been exposed to is a straight forward way of conceptualising and measuring CM, however it does not reflect the fact that categories of CM or ACE are not equal in their severity or impacts [70]. Analyses based on specific exposures to sub-types of CM or ACE can reflect differential severity and impacts of different types of maltreatment, but fail to reflect the common clustering of maltreatment types (for example intra-familial sexual abuse will almost always be association with physical abuse, emotional abuse, and neglect), and can lose this inherent complexity [70]. Recent work using latent class analysis has identified common clusters of childhood adversity (such as household dysfunction, parental loss, maltreatment and conflict, and polyadversity) which may represent a better way of conceptualising this area moving forwards [71,72]. Furthermore it is important to recognise and account for wider forms of adversity which are not fully reflect in traditional conceptions of CM and ACE (which focus more on the immediate family environment), in particular socioeconomic status and wider social adversities such as discrimination [72,73]. In a similar vein, neurodevelopmental conditions are related to risk of exposure to CM [74] but were not considered as covariates in any of the included studies.

This review focused on peripheral measures of inflammation as this is the most widely studied in this field. Peripheral measures of inflammation have been found to correspond to central nervous system inflammation [18,19], however a recent meta-analysis of inflammation in depression found that central measures of inflammation as measured by cerebrospinal fluid sampling, and positron emission tomography did not correlate with peripheral measures of inflammation [75]. Studies of CM and inflammation have generally measured the association between CM and a single biomarker (particularly CRP and IL-6) as indicative of inflammation. Del Giudice and colleagues have noted that biomarkers such as CRP and IL-6 have wider biological functions beyond the inflammatory response and that elevations in these biomarkers are not necessarily reflective of inflammation [76]. Inflammation may be better measured by multiple biomarkers over time. Of note in this review only one study measured inflammation (CRP) over time [54]. Included studies did attempt to exclude the impact of acute inflammation by excluding participants with markedly elevated CRP, however a single measure of an inflammatory marker may not be an accurate measure of a chronic inflammatory state. Furthermore a meta-analysis of the association between early life adversity (ELA) and inflammation in under 18s demonstrated different patterns of association in differently aged samples (eg. ELA is associated with inflammation in studies of infants and adolescents but not children) [26]. This suggests that the association between ELA and inflammation is not consistent across the lifespan. Longitudinal measures of inflammation in adults would help identify any such trajectories across adulthood. Of note in our review a clearer association between CM and inflammation was evident in studies of older adults.

Studies varied in their accounting for potential confounding and mediating variables. Of note, BMI appears to have an important role in the relationship between CM, systemic inflammation, and psychopathology. As highlighted previously, most studies finding significant associations between CM and systemic inflammation did not adjust for BMI or related measures (e.g. waist-hip ratio), yet studies employing structural equation modelling suggested that the relationship between CM and inflammation might be mediated by BMI. Based on the current literature it is plausible to speculate that the association between CM and systemic inflammation might be primarily mediated by elevated BMI, but further direct data on this possible association are required.

Most studies were based in Europe or North America and predominantly included participants of white ethnicity. One study [39] found a significant interactions with ethnicity, finding a relationship between neglect and CRP in people of white ethnicity only [39]; a further study of early life adversity and inflammation (not included due to exposure being wider adversity), contrastingly found early adversity to be associated with IL-6, fibrinogen, E-Selectin, and sICAM for African-Americans only [77]. Associations between ethnicity and health outcomes are likely to be confounded by a range of social and environmental factors [73], particularly in the USA, where there are strong associations between ethnicity and poverty and poor access to healthcare and it would be helpful for these apparent associations to be explored more widely and in other settings.

None of the included studies directly examined the role of gender, however many studies were conducted exclusively in females, or in predominantly female sample which may impact on the overall results. Of note one study of the association of early adversity with inflammation (not eligible for inclusion in this review due to inclusion of wider adversities) found a significant relationship between adversity and CRP in females only, suggesting that gender may be an important factor in understanding the relationship [78].

This systematic review is subject to several limitations. Whilst attempts were made to be exhaustive, practical limitations precluded inclusion of foreign language titles and grey literature. The original protocol for this study aimed to perform a meta-analysis but this was unfortunately neither practicable nor appropriate due to: i. significant variation in the exposure concept (CM as a dichotomous or continuous variable; as an overall construct or as sub-components), ii. the use of various measurement tools (in particular, difficulties combining between- group comparisons and linear analyses), and iii. inconsistencies in adjustment for covariates where this was done at all. These problems would have significantly impacted the statistical robustness of any findings and potentially created more confusion or, worse, amplified biases in this already challenging field. In the absence of comparable statistical measures of effect, it was not feasible to formally assess for publication bias. Subjectively, there is no clear association of study size with statistically significant results which would point against significant publication bias, however this cannot be excluded.

Overall this systematic review has identified an association between CM and elevated CRP in prospective studies, however findings of retrospective studies and for other biomarkers are conflicting. Tentatively at least part of the association between CM and systemic inflammation may be mediated by the association between CM and elevated BMI, which itself may be driven by physiological (such as dysregulated stress-reactivity leading to dysregulation of metabolic pathways) or psychological (such as emotional dysregulation or impulsivity leading to dysregulated eating behaviours) factors, or indeed both. Obesity is strongly associated with low-grade inflammation in a mechanism which may be partially mediated by alterations in the gut microbiome and gut permeability [79], factors which have also been suggested as important drivers of low-grade inflammation and age-related disease [80]. Additional previously unmeasured covariates may also mediate the association of CM with elevated BMI and inflammation, for example neurodevelopmental disorders (which previous work by our group has shown to be associated with obesity [81]) and the gut microbiome, which may mediate the relationship between a range of adverse exposure and inflammation [80]. All of this highlights the importance of applying complex systems methodologies to exploring the interaction of variables holistically and longitudinally [82].

Achieving the research goal of understanding these potentially complex mechanisms would have practical relevance since, if the main mediator is obesity or the gut microbiome, the most effective interventions would likely involve weight loss, exercise, dietary change and early intervention to prevent obesity; whereas if the association between CM and systemic inflammation were more direct, this may point towards a role for anti-inflammatory medications. Further prospective, longitudinal, research using robust and comparable measures of CM with careful consideration of confounding and mediating variables, particularly BMI, are required to bring clarity to this field.

## Supporting information

S1 Table Details of excluded articles. (DOCX)

S2 Table Results relating to other biomarkers. (DOCX)

S1 File Details of search strategy. (DOCX)

S2 File PRISMA checklist. (DOCX)

S3 File Systematic review protocol. (PDF)
