# Supplementary material for: The association of child maltreatment and systemic inflammation in adulthood: A systematic review
Source: PLoS One. 2021 Apr 8;16(4):e0243685. doi: 10.1371/journal.pone.0243685 (PMC8031439; doi:10.1371/journal.pone.0243685)
Supplement: S1 Table — (DOCX) [file pone.0243685.s001.docx]

S1 Table- Articles reviewed in full and excluded:

| Article | Reason for Exclusion | Details |
| --- | --- | --- |
| Altemus M *et al*. Am J Psychiatry 2003; 160: 1705-1707 | No direct comparison of child maltreatment with control | PTSD (secondary to childhood abuse) vs control. Analysis not specific to effects of childhood maltreatment. |
| Appleton *et al* Health Psychology 2012; 31(4): 413-422 | ACEs | Exposure is socio-economic adversity not child maltreatment |
| Archer *et al* Journal of Affective Disorders 2012; 143: 39-46 | Inflammatory condition | Limited to participants with cancer |
| Ayaydin H *et al*. Paediatrics International 2016; 58: 105-112 | No direct comparison of child maltreatment with control | PTSD (secondary to child sexual abuse) vs control. Analysis not specific to effects of childhood maltreatment. |
| Baldwin *et al* Brain, behavior, and immunity. 2018;67:211-7 | ACEs | Exposure includes bullying and exposure to parental domestic violence |
| Bellinger *et al* Journal of Immunotoxicology 2008; 5(4): 419-444 | Review article | Review article |
| Berens *et al* BMC Medicine 2017; 15(1): 135 | Review article | Review article |
| Bick *et al* Dev Psychobiol 2015; 57(1): 131-139 | <18s | <18s |
| Bielas *et al* Journal of Psychosomatic Research 2012; 73: 313-318 | <18s | <18s |
| Bower *et al* Childhood maltreatment and monocyte gene expression among women with breast cancer. Brain Behavior and Immunity 2020- In press | Gene expression | Outcome is gene expression not blood based biomarkers. Also limited to participants with cancer. |
| Bublitz *et al* Obstetric Medixine 2017; 10(3): 120-124 | No direct comparisons | Reports effect over time only, no baseline comparisons. |
| Bucker J *et al* Acta Psychiatrica Scandinavica 2015; 131: 360-368 | No comparison | PTSD (secondary to childhood abuse) vs control. Analysis no specific to effects of abuse/neglect. |
| Carpenter *et al* Neuropsychopharmacology 2010 35: 2617-2623 | Stress testing | Trier Social Stress Test |
| Carroll J *et al*PNAS 2013; 110 (42): 17149-17153 | Composite score | Measures CRP and Il-6 as part of a multi-systemic risk score. Values dichotomised as highest risk and aggregated into this score. No comparisons of CRP or Il-6 exposure vs control so cannot include. Of interest abuse/neglect associated with higher multi-systemic risk scores but significant protective interaction with parental warmth. |
| Chen *et al* Brain, Behaviour, and Immunity 2018; 69: 582-590 | ACEs | Exposure is ACEs, not specifically child maltreatment. |
| Cho *et al* Brain Behavior and Immunity 2012; 26(6): 859-865 | ACEs | Broader adversity including parental substance use and mental illness. |
| Cichetti *et al* Dev Psychobiol 2015; 27(2): 553-566 | <18s | <18s only |
| Cohen-Woods *et al*. Brain 18; 67: 203-210 | Genetics | Genetic outcome only |
| Crosswell *et al* Psychosom Med 2014; 76(3): 208-214 | Inflammatory condition | Limited to participants with cancer. |
| Danese *et al*; Arch Gen Psychiatry 2008; 65(4): 409-415 | Duplicate sample | Reports on same Dunedin sample as Danase 2007. Relevant results show elevated CRP in MDD + child maltreatment vs controls but NS elevation in MDD no maltreatment vs controls. |
| Danese *et al* Arch Pediatr Adolesc Med 2009 163(12): 1135-1143 | Duplicate sample | Reports on same sample as Danase 2007. |
| Danese *et al* Mol Psychiatry 2011; 16(3): 244-246 | <18s | <18s |
| Danese *et al* Physiology and Behavior 2012; 106(1): 29-39 | Review article | Review Article |
| Danese *et al* Translational Psychiatry 2014; 4(9): e446 | <18s | <18s |
| David *et al* Developmental Psychobiology 59(6): 696-702) | <18s | <18s (infants) |
| Deschenes *et al* 2018; 41: 2120-2126 | ACEs | Exposure is wider ACEs not child maltreatment |
| Di Nicola *et al*  Brain, Behavior and Immunity 2013; 31: 90-95 | ACEs | Broader adversity including parental loss and separation. |
| Dich *et al*. Anxiety stress and coping 2015; 28(4): 372-390 | Composite outcome | Outcome is allostatic load not specific for CRP. Exposure is broad adversity. |
| Do Prado *et al* Developmental Psychobiology 2016; 58(4): 482-491 | Animal model | Animal models |
| Do Prado. *Et al* Neuropsychopharmacology 2017; 42*11): 2272-2282 | <18s | <18 |
| Druzhkova *et al*  Neuroscience and Behavioural Physiology 2019; 49(7): 916-920 | No direct comparison of child maltreatment with control group | No specific analyses of relationship of abuse with inflammation. |
| Druzhkova. *Et al* Zhurnal Nevrologii I Psihiatrii imeni S.S. Korsakova 2018; 118(6): 10-14 | Non-English | Not in English (Russian). Full text identified (above) and not meeting criteria for inclusion. |
| Ehrlich *et al* Development and psychopathology 2016; 28(4): 1273-1283 | <18s | <18s |
| Elwenspoek *et al* Journal of Immunology 2017; 199(12): 4046-4055 | ACEs | ACEs |
| Elwenspoek *et al* Psychoneuroendocrinology 2017; 82: 140-154 | Review | Review |
| Friedman *et al* Psychosom Med 2015; 77(2): 176-185 | Composite score | Outcome allostatic load. No specific results for inflammatory markers. Exposure broader adversity not specifically abuse/neglect. |
| Gallo *et al* Psychosomatic Medicine 2019 81(3): 305-312 | ACEs | Exposure is socio-economic adversity |
| Geiger *et al* Mental Health and Prevention 2019; 13: 176-186 | In vitro measures | In vitro measures only |
| Ghosh *et al* PloS One 13(6): e0198412 | Broader ACEs | Exposure “chronic sexual abuse” combining child and adult exposure. No specific findings for CSA. |
| Gill *et al* Journal of Affective Disorders 2020; 272: 1-7 | Review | Review |
| Gill *et al* Journal of Traumatic Stress 2008; 21(6): 530-539 | No direct comparison | No specific comparisons of child maltreatment versus control. |
| Grosse *et al* Brain, Behaviour and Immunity 2015; 44: 48-56 | Gene expression | Monocyte gene expression only |
| Han *et al* Psychooncology 2016; 25(2): 187-193 | Inflammatory condition | Reports exclusively on inflammation in breast cancer with radiotherapy treatment |
| Heim *et al* Psychoneuroendocrinology 2008; 33(6): 693-710 | Review | Review |
| Hicks *et al* Journal of the American Association for Laboratory Animal Science 2017; 56(5): 636 | Animal model | Animal Model |
| Holland *et al* Social Psychiatry and Psychiatric epidemiology 2020 | <18s | <18s |
| Hostinar *et al* Developmental Psychology 2015; 51: 1630-1644 | ACEs | Exposure in ACEs not specifically child maltreatment |
| Hostinar *et al* Psychoneuroendocrinology 2017; 75: 152-163 | No direct comparison. | Predicting inflammation from frontal brain asymmetry on EEG. No direct results of maltreatment to inflammation. Composite inflammatory marker, no individual markers. |
| Irwin *et al* Neuropsychopharmacology 2019; 44(3): 635-641 | Stress testing | Stimulated response to endotoxin. Gene expression rather than blood markers. |
| Janusek *et al*; Brain Behaviour Immunity 2013; 30(Suppl): S149-S162 | Inflammatory condition | Reports exclusively on inflammation in breast cancer with radiotherapy treatment. |
| Janusel *et al* Brain, Behavior, and Immunity 2017; 60: 126-135 | Stress testing | Trier social stress test only |
| John-Henderson *et al* Ann. Behav. Med 2020; 54: 87-93 | ACEs | Exposure is ACEs. |
| Johnson *et al*; Development and Psychopathology 2017; 29: 1935-1946 | Composite outcome | Reports on composite cardiometabolic risk which includes CRP. Doesn’t specifically report on CRP. |
| Joung *et al* J Clin Endocrinol Metab 2014; 99(6): E1055-1060 | ACEs | Early life adversity not specific abuse/neglect and no breakdown by sub-types of adversity. |
| Konradt *et al* Rev Psiq Clin 2013; 40(3): 93-6 | No inflammatory markers | No inflammatory markers reported |
| Kraav *et al* Psychological Medicine 2020 1-3 | ACEs | ACEs |
| Kuras *et al* Dev Psychibiol 2017; 59: 91-98 | Stress testing | Trier social stress testing |
| Lacey *et al* Brain, Behaviour, and Immunity 2020- In Press | ACEs | Exposure is ACEs. More focused on interaction of ACEs. |
| Lacey *et al* Psychoneuroendocrinology 2013; 38(11): 2476-2484 | ACEs | Exposure is parental separation |
| Lemieux *et al* Brain, Behav, Immun 2008; 22(6): 994-1003 | No direct comparison of child maltreatment and control | Focused on PTSD and association of T-cell activation with PTSD symptom behaviour |
| Levandowski *et al* Psychiatry Research 2013; 210: 536-540 | Stress response | Inflammatory response to crack cocaine withdrawal. Not comparable with other studies. |
| Levandowski *et al* Drug & Alcohol Dependence 2016; 167: 140-148 | Stress testing | Inflammatory response to crack cocaine withdrawal. Not comparable with other studies. |
| Li *et al* BMC Geriatrics 2015; 15: 102 | ACEs | Exposure is broader adversity |
| Lin *et al* Brain Behav Immun 2016; 53: 105-112 | ACEs | Exposure childhood adversity (repeating a year of school, parental substance misuse, and/or physical abuse). No specific results for abuse. |
| Lin  *et al* Brain Behav Immun 2016; 53: 105-112 | ACEs | Exposure is broadly defined adversity- repeating year at school, parental substance misuse, and abuse. Doesn’t separate in analysis. |
| Lopes *et al* NeuroImmunoModulation 2011; 19(1): 33-42 | In vitro measure | *in vitro* measures not blood biomarkers. |
| Marsland *et al* Psychosom Med 2013; 75(5): 438-41 | Review | Review Article |
| Matthews.*et al* Psychosomatic Medicine 2016; 78(3): 311-318 | ACEs | Exposure is childhood socio-economic status not abuse. |
| McDade *et al* Brain Behavior and Immunity 2013; 31: 23-30 | ACEs | Exposure is broader adversity- parental separation and socioeconomic adversity. |
| McQuaid *et al* Frontiers in Psychiatry 2019; 10:151 | Genotypes | Il-1b genotypes as moderator between CTQ and depression. Not measuring inflammatory marker. |
| Menke *et al* Psychoneuroendocrinology 2018; 98: 22-29 | No inflammatory markers | HPA axis not specifically inflammatory markers. |
| Miller *et al* Biological Psychiatry 2012; 72(1): 34-40 | <18s | <18s, exposure is broader adversity |
| Mitchell *et al* Health Psychol 2018; 37(2): 114-124 | No direct comparisons | Effects over time (rates of change through pregnancy). No direct comparisons of particular timepoint. |
| Muller *et al* Journal of Child and Adolescent Trauma 2014; 7(3): 185-191 | <18s | <18s, stress testing only |
| Nettle *et al* Scientific Reports 2017; 7: Article No. 40794 | Animal Model | Animal model |
| Neuroscientist 2008; 14(5) 405 | Review | Review article |
| Orso *et al* Neuroscience letters 2017; 658: 27-31 | Animal | Animal model |
| Pace *et al* Americal Journal of Psychiatry 2006; 163(9): 1630-1633 | Stress testing | Trier social stress testing |
| Pace *et al* Brain Behavior and Immunity 2012; 26(1): 13-17 | Gene expression | Outcome is gene expression (NFKB) not peripheral biomarker. |
| Peters *et al* Journal of Psychiatric Research 2019; 114: 1-10 | <18s | <18s |
| Petrov *et al* J Behav Med 2016; 39(4): 716-726 | No direct comparison of child maltreatment and control | Exploring inflammation as mediator between abuse and hypertension, no direct results on abuse and inflammation |
| Powers *et al* Comprehensive Psychiatry 2019; 93: 33-40 | No direct comparison of child maltreatment and control | Appears to be reporting on same sample as Powers 2016. This is more focused on PTSD and dissociation rather than specific association of childhood abuse/neglect with inflammation |
| Raposa *et al* Psychological Science 2014; 25(6): 1268-1274 | ACEs | Exposure is ELS defined by maternal mental illness, low family income, harsh discipline, parental discord, or parental incarceration- not abuse/neglect. |
| Rasmussen *et al* 2020 JAMA Pediatr; 174(1): 38-47 | ACEs | Broader adversity including domestic violence, crime, bullying etc. |
| Rasmussen *et al* Journal of Child Psychology and Psychiatry 2019 60:2 199-208 | ACEs | Broader adversity |
| Reid *et al*  Brain Behavior and Immunity 2019; 77: 168-177 | <18s | <18s |
| Reid *et al* Brain, Behavior, and Immunity. 2020; 86: 4-13 | <18s | <18s |
| Reus *et al* Journal of Psychiatric Research 2017 95: 196-207 | Animal model | Animal models |
| Rohleder *et al* Psychosomatic Medicine 2014; 76(3): 181-189 | Review | Review |
| Runsten *et al* Nordic Journal of Psychiatry 2014; 68(2): 137-144 | ACEs | Broader adversity |
| Russell *et al* Journal of Child Psychology and Psychiatry 2019; 60:10 1094-1103 | ACEs | Broader adversity |
| Saban *et al* Brain Behaviour Immunity 2018; 73: 625-623 | ACEs | Main exposure is perceived discrimination. CTQ is a covariate with no direct results presented. |
| Serbulent *et al* Saudi Med J 2017; 38(12): 1213-1218 | <18s | <18s. |
| Shirtcliffe. *Et al* PNAS 2009; 106(8): 2963-2967 | <18s | <18s |
| Simons *et al* Journal of Youth and Adolescence 2019; 48(1) | ACEs | Exposure is broadly defined adversity |
| Slopen *et al* Psychosomatic Medicine. 2010;72(7):694-701 | ACEs | Exposure is broadly defined adversity |
| Slopen *et al* Psychoneuroendocrinology 2015; 51: 403-413 | ACEs | Exposure is broadly defined adversity |
| Stadler *et al* Brain Behavior and Immunity 2020- In Press | Review | Review Article |
| Surtees *et al* International Journal of Behavioural Medicine 2003; 10(3): 251-268 | ACEs | ACEs only. |
| Takizawa *et al* Psychological Medicine 2015; 45: 2705-2715 | ACEs | Exposure bullying not abuse/neglect. Controls for adversity. No specific figures for abuse/neglect. |
| Taylor *et al* PNAC 2010; 107(19): 8507-8512 | Review | Review |
| Taylor *et al* Biological Psychiatry 2006; 60(8): 819-824 | ACEs | Exposure is wider adversity |
| Thayner. *Et al* Am J Hum Biol 2017; 29(3): | ACEs | Exposure is broadly defined adversity. Outcome is allostatic load. |
| Tietjen *et al* Headache 2012; 52: 920-929 | ACEs | Exposure is ACEs |
| Van Ockenburg *et al* Acta Psychiatrica Scandinavica 2015; 131: 40-50 | ACEs | Exposure is broadly defined life events. |
| Wilson *et al* Psychosomatics 1999 40(3): 222-225 | No direct comparison of child maltreatment vs controls. | Exclude- PTSD from abuse vs controls. No specific effect of child maltreatment. |
| Woods *et al* Advances in Nursing Science 2015; 28(4): 306-319 | No direct comparison of child maltreatment vs controls. | PTSD from childhood trauma or adult violence. |
| Zeugmann *et al* Psychiatrica Danubina 2012; 24(1): 57-65 | No inflammatory markers | Metabolic syndrome, not inflammatory markers |
