# Supplementary material for: The association of child maltreatment and systemic inflammation in adulthood: A systematic review
Source: PLoS One. 2021 Apr 8;16(4):e0243685. doi: 10.1371/journal.pone.0243685 (PMC8031439; doi:10.1371/journal.pone.0243685)
Supplement: S2 Table — (DOCX) [file pone.0243685.s002.docx]

S2 Table- Findings relating to other biomarkers

| Citation | Study characteristics | Sample Size | Sample Demographics | Measure of abuse/neglect | Biomarkers | CCAT | Main Findings |
| --- | --- | --- | --- | --- | --- | --- | --- |
| Aas 2017 [1] | Participants in TOPS study of psychotic disorders in Oslo Norway. Consecutively recruited outpatients with schizophrenia and bipolar disorder and matching controls | 483 | Schizophrenia:  Age= 28.6 (9.3)  Female= 55 (41.0%)  BMI= 26.3 (5.5)  Bipolar Disorder  Age= 32.2 (11.7)  Female= 73 (59%)  BMI= 25.8 (4.3)  Control:  Age= 30.9 (7.5)  Female= 86 (41%)  BMI= 24.0 (3.4) | CTQ subcomponents; dichotomised | CRP, gp130, sTNFR1 | 36 | No significant effect of CM on gp130 and sTNFR-1. |
| Bertone-Johnson 2012 [2] | Participants in Nurses Health Study II. A cohort of female nurses in USA recruited in 1989. A sub-sample was invited to provide blood samples between 1996-1999. | 702. | Age= 48.9 (SD not reported)  Female= 100%  BMI= 25.8 (SD not reported) | Physical abuse- Adapted Revised Conflict Tactics Scale.  Sexual abuse- Adapted Sexual Experiences Survey | CRP, Il-6, sTNF-R2 | 33 | sTNF-R2 was not associated with any measure of PA or SA in unadjusted or adjusted models. |
| Corsi-Zuelli 2020 [3] | Participants in the STREAM Cohort of patients with first episode psychosis, unaffected siblings, and healthy controls in Sao Paulo Brazil. | 422 | FEP:  Age= 30.8 (12.5) Female= 37% BMI= 24.8 (5.1)  Siblings:  Age= 30.7 (10.5) Female= 68.4% BMI= 24.9 (4.9)  Controls:  Age= 31.3 (11)  Female= 48.6%  BMI= 26.2 (5.3) | CTQ- subcomponents; dichotomised | Il-1b, Il-4, Il-6, Il-10, TNF-a, TGF-b, IFN-y | 35 | In analyses adjusted for age, gender, BMI, smoking, substance use, education, and relationship status, only significant effect of abuse exposure was for TGF-b.  Of CTQ sub-components only PA was associated with TGF-b. On post-hoc analysis in FEP group PA associated with higher TGF-b. No difference in siblings by abuse. In controls PA associated with lower TGF-b. |
| Counotte 2019 [4] | Participants in a study of participants with psychotic disorders, ultra-high risk for psychosis, unaffected siblings, and controls, in the Netherlands. | 117 | Psychosis  Age= 25.5 (23-30)  Male= 6 (15.8%)  BMI=23.0 (20.4-24.3)  UHR  Age= 24.0 (20-29)  Female= 6 (54.5%)  BMI= 23.1 (18.5- 26.2)  Siblings  Age= 25.5 (21.3- 30.0)  Female= 12 (41.4%)  Controls:  Age= 24.0 (21.0-26.0)  Female= 21 (53.8%) | CTQ- total; dichotomised | BDNF, chemokine ligand-2, IGFBP2, PDGF, SCF, TNF-a, Il-6, CRP | 38 | No significant effect on CTQ on any inflammatory marker in models which adjust for age, sex, BMI, smoking, drug use, and education. |
| Danese 2007 [5] | Participants in longitudinal cohort study in Dunedin New Zealand. Bloods collected at visit at age 32. | 892. | Age= 32  Female= 435 (48.8%) BMI not stated | Prospectively recorded childhood maltreatment including parental rejection and harsh discipline, and retrospective report of physical and sexual abuse.  Scored as no (64%), probable (27%) and definite (9%). | CRP, fibrinogen, WCC | 35 | Childhood maltreatment associated with elevated CRP, WCC, and fibrinogen, in unadjusted analyses and models adjusting for childhood confounders, and adult health and behaviour, including overweight.  Estimates that 10% of low-grade inflammation as measured by CRP may be attributable to childhood maltreatment. |
| Dennison 2012 [6] | Patients with schizophrenia in Cork Ireland recruited from outpatient and inpatient settings. Controls recruited from local university. | 80 | Schizophrenia  Age= 38.33 (1.7)  Female= 16 (40%)  BMI= 84.01 (2.88)  Controls:  Age= 36.2 (1.76)  Female= 27 (67.5%)  BMI=23.89 (0.83) | CTQ- total; dichotomised | Il-1b, Il-6, Il-8, TNF-a | 31 | Compared differences in cytokines across groups (schizophrenia with abuse, schizophrenia without abuse, and control) using ANOVA. Significant group differences for Il-6 and TNF-a only. |
| Grassi-Oliveira 2009 [7] | Female outpatient with MDD in partial and controls recruited from hospital staff in Brazil. | 49 | MDD:  Age= 39.21 (8.56) Female= 100% BMI= 26.89 (2.86)  Control Age= 37.36 (5.48) Female= 100% BMI= 25.54 (2.50) | CTQ- total; continuous | sTNFR1, sTNFR2, TNF-a- not detectable so not included in analysis | 34 | In total sample significant correlation of CTQ and sTNFR2. No significant correlation with sTNFR1. |
| Hartwell 2013 [8] | Control group in a larger study on gender differences in stress responses in cocaine dependency in South Carolina USA. Participants were non-cocaine using controls with no mental or physical illnesses. | 39 | Age= 35.69 (12.0)  Female= 20 (51.3%) BMI= 26.8 (4.3) | ETI- Number of traumas | Il-1b, Il-6, TNF-a, CRP | 36 | Number of traumas was significantly associated with elevated Il-1b, in models adjusted for age, sex, and smoking status.  In analysis of subscales general trauma only was associated with elevated Il-1b. |
| Imai 2008 [9] | Female outpatients with PTSD and matching controls in Japan. Participants had no significant physical illnesses. Analysis of inflammation and abuse only included the PTSD group. | 40 (participants with PTSD) | Age= 38.3 (10.1)  Female= 100%  BMI= 22.0 (3.5) | CTQ- total; continuous | Il-1b, Il-6, TNF-a, CRP, sIL-6R | 37 | No correlation of CTQ (total or subscales) with any inflammatory marker in unadjusted analysis. |
| Kim 2019 [10] | Healthy students at a university in Texas USA. | 85 | Age=21.0 (no SD)  Female= 61 (71.7%) BMI not stated | ACEs questionnaire- specific analyses based on items on abuse and neglect | BDNF, CRP | 29 | No significant association of abuse or neglect with BDNF. |
| Lehto 2012 [11] | Participants in the KUDEP study of depression in the general population in Kuopio Finland. This study was limited to participants with a history of “adverse mental symptoms”- low mood (BDI), alexythmia (TAS), and reduced life satisfaction (LSS) | 147 | Maltreatment  Age= 54.5 (46.75-60.00)  Female= 18 (60.0%)  BMI=26.85 (25.40-29.95)  Control:  Age= 56.00 (49.00-63.00)  Female= 68 (58.1%)  BMI= 27.10 (23.60-30.45) | Derived from items of baseline questionnaire. Childhood maltreatment consisted of presence of physical and/or sexual abuse. | Apidonectin, resistin | 30 | In a model adjusted for age, gender, and BMI lower levels of adiponectin was associated with increased odds of being in the abuse group (by logistic regression).  There was no association of childhood maltreatment and resistin. |
| Lu 2013 [12] | Study of outpatients with MDD (with and without and history of childhood maltreatment) and controls recruited from Changsha China. Participants had no other mental or physical illnesses. | 65 | MDD and CM:  Age= 30.2 (8.53)  Female= 14 (63.6%)  BMI=21.1 (2.11)  MDD no CM:  Age= 30.1 (6.81) Female= 11 (52.3%)  BMI= 21.8 (2.35)  Control:  Age= 27.7 (4.78)  Female= 11 (50%)  BMI= 21.8 (2.05) | CTQ- total; dichotomised | 120 cytokine array (only 13 reported:  - AgRP, bFGF, BTC, GITR-L, I-TAC, Il-1b, Il-1R1, MEC, NT-4, TECK, TGF-b, TRAIL-R4, VEGF | 24 | No correlation of CTQ with any cytokine in MDD and CM group. |
| Pedrotti Moreira 2018[13] | Cross-sectional study of participants with MDD in Pelotas Brazil. Participants had no other mental or physical illness. | 166 | Age= 25.87 (5.25)  Female= 124 (74.7%)  BMI= 26.03 (5.41) | CTQ- total; continuous | Il-6, Il-10, TNF-a | 34 | In depressed participants abuse was associated with elevated Il-10. This remained significant on linear regression adjusting for schooling and smoking status. |
| Muller 2019[14] | Inpatient and outpatients with MDD and matching controls (from local university) recruited in Munich Germany. No other mental or physical illnesses. | 88 | MDD  Age= 39.2 (12)  Female= 17 (39%)  BMI=23.6 (3.2)  Control  Age= 38.9 (11.8)  Female= 17 (39%)  BMI=24.1 (4.1) | CTQ- subcomponents; continuous | Il-6, Il-10 | 35 | In total sample no association of Il-10 with abuse sub-types.  In control group SA correlated with Il-10 only. |
| Pinto Pereira 2019[15] | 1) 1958 British Birth Cohort: A longitudinal cohort of all born in one week in 1958. Bloods collected at visit age 45.  2) Midlife in United States (MIDUS): Cohort of adults 25-75 in USA recruited in 1994-1995. 2^nd^ wave of data collection (MIDUS-II) followed up original sample 10 years later. | British Birth Cohort= 7661  MIDUS= 1255 | British Birth Cohort:  Age= 45.2 (44.3-46.0) (mean and range)  Female= 3828 (50%)  BMI=  - Males= 27.7 (4.18) - Female= 26.9 (5.48)  MIDUS Age - Male= 57.9 (36-86) - Female= 56.9 (35-86)  Female= 713 (56.8%)  BMI=  - Male= 29.7 (5.38) - Female= 29.9 (7.44) | Physical, emotional and sexual abuse; emotional neglect and physical neglect (British Birth Cohort only).  Derived from study questions. All measures retrospective except from prospective record of neglect in British Birth Cohort. | CRP Fibrinogen Il-6 (MIDUS only) | 25 | In linear regression adjusted for age, race, gender, and other types of abuse, physical abuse was associated with increased fibrinogen in BBC only.  Other forms of maltreatment were not associated with fibrinogen in either sample. |
| Porcu 2018[16] | Women aged 18-65 in Londrina Brazil. Participants were patient with unipolar or bipolar depression recruited from outpatient clinics, non-depressed smokers were recruited from smoking cessation clinics and non-smoking controls from hospital staff. Participants excluded if had other mental or physical illness | 159 | Depressed Smokers:  Age= 46.07(10.58)  Female= 100%  BMI= 26.49 (5.07)  Depressed Never-Smokers:  Age= 41.08 (13.61)  Female= 100% BMI= 29.34 (6.63)  Control Smokers:  Age= 43.54 (11.85) Female=100% BMI= 28.18 (6.05)  Control never-smokers:  Age= 39.21 (13.19)  Female=100% BMI= 24.73 (3.47) | CTQ- subcomponents; continuous | Il-1b, Il-6, Il-10, Il-12, sTNF-R1 | 23 | In depressed smokers only, emotional neglect was correlated with sTNF-R1.  There were no other significant associations with maltreatment and inflammatory markers. |
| De Punder 2018[17] | Patients with depression were recruited from an affective disorders clinic and controls from public advertisements. Participants did not have other mental or physical illnesses. Neither patients or controls taking psychotropic medications. Study in Berlin Germany | 86 | MDD and MT Age= 38.09 (11.36)  Female= 14  BMI=25.05 (2.93)  MDD no MT  Age= 32.61 (11.74) Female= 18  BMI= 25.41 (8.96)  Control and MT  Age= 38.09 (11.36) Female= 14  BMI= 23.79 (3.14)  Control no MT:  Age= 33.9 (9.77)  Female= 13  BMI= 23.23 (3.56) | ETI- Total; Dichotomised | CRP, Il-6, WCC | 31 | Significant difference between groups for WCC which remained significant after adjusting for BMI and smoking. |
| Slopen 2013[18] | Participants in Add Health Cohort following adolescents from high school to early adulthood in a representative sample from USA. | 13244 | Age= 28.95 (0.12)  Female= 50.53%  BMI not stated | No formal tool used. Asked retrospectively about PA and SA. Included details on timing and frequency | Cell-mediated immunity as determined by EBV antibody titre.. | 30 | Overall PA and SA not associated with EBV titres.  SA frequency >10x associated with elevated antibodies vs no abuse.  PA beginning in preschool period associated with elevated antibodies vs no abuse. |
| Smith 2011[19] | Participants in a larger study of influence of genetics and environmental responses to stress in African Americans in Atlanta Georgia. Participants grouped by presence of PTSD and early maltreatment  Also describe “extended sample” of 177- unclear origin | 110  34 | Sample demographics not reported. States that groups were matched for age and gender. | CTQ- Total; dichotomised | Il-1b, Il-2, Il-4, Il-6, Il-10, IFN-a, TNF-a | 27 | Maltreatment associated with TNF-a but no other inflammatory markers. |
| Thurston 2017[20] | Participants in the MsHeart study of peri- and post-menopausal women aged 40-60 in Pitsburgh USA. Excluded if history of signficiant physical illness | 286 | Abuse/Neglect  Age= 53.68 (4.11) Female= 100% BMI= 29.11 (7.50)  No abuse/neglect:  Age= 54.28 (3.90)  Female= 100%  BMI= 28.88 (6.07) | CTQ- total and subcomponents; dichotomised | CRP, Il-6, Fibrinogen, vWBF, D-dimer | 29 | No significant differences between groups for any biomarker. |
| Toft 2018[21] | Patients with MDD admitted to a psychiatric hospital in Norway. | 128 | Mild:  Age= 39.4 (10.8) Female= 20 (71.4%)  Moderate  Age= 44.2 (12.0) Female= 28 (66.7%)  Severe  Age= 41.3 (11.7)  Female= 44 (75.9%) | No formal measure. Asked about trauma on admission interview and grouped as trauma present or absent. | Il-1b, Il-1RA, TNF-a, MCP-1 | 27 | Trauma was significantly associated with higher levels of Il-1RA and TNF-a only. |
| Waldron 2016[22] | Healthy female college students in Virginia USA | 89 | Age= 19.24 (1.29)  Female= 100%  BMI not recorded | Child Abuse Survey- Modified. Grouped as no abuse, physical abuse and sexual abuse | Salivary IgA | 27 | No association of any abuse type with salivary IgA. |
| Zeugmann 2013[23] | Inpatients with MDD in Germany. | 25 | Age= 47.8 (15.02)  Female= 68% | CTQ- Subcomponents; dichotomised | Fibrinogen, SAA, CRP, adiponectin, TNF-a, resistin, sE-selectin | 31 | Only significant association between physical neglect and fibrinogen. |

Legend: AgRP: Agouiti related protein, BDNF: Brain derived neurotropic factor, bFGF: Basic fibroblast growth factor, BMI: Body mass index, BTC: betacellulin, CCAT- Crowe Critical Appraisal Tool, CL-2: Chemokine-Ligand 2, CRP: C-reactive protein, CTQ: Childhood trauma questionnaire, EBV: Epstein-Barr Virus, EA: Emotional abuse, EN: Emotional neglect, ETI: Early trauma inventory, IFN-y: Interferon gamma, GITR-L: Glucocorticoid induced tumour necrosis factor ligand, gp130: glycoprotein 130, IGFBP2: Insulin-like growth factor-binding protein 2, I-TAC: Interferon induced T-cell alpha chemoattractant, Il-1RA: Interleukin-1 Receptor Antagonist, Il-1b: Interleukin 1 beta, Il-2: interleukin-2, Il-4: Interleukin-4, , Il-6: Interleukin-6, Il-8: Interleukin-8, Il-10: Interleukin-10, Il-12: Interleukin-12, MCP-1: Monocyte chemoattractant protein-1, PA: Physical abuse, PDGF: Platelet derived growth factor, PN: Physical neglect, SA: Sexual abuse, sAA: salivary alpha amylase, SCF: stem cell factor, sICAM2: soluble intracellular adhesion molecule 2, sIl-6R: soluble interleukin-6 receptor, sTNFR1: soluble Tumour Necrosis Factor Receptor 1, sTNFR2: soluble Tumour Necrosis Factor Receptor 2, TECK: thymus expressed chemokine, TGF-b: Transforming growth factor beta, TNF-a: Tumour Necrosis Factor Alpha, TRAIL-R4: Tumour necrosis factor related apoptosis induced ligand-receptor 4, VEGF: vascular endothelial growth factor, vWF: von Wilibrand Factor, WCC: White cell count

References

1. Aas M, Dieset I, Hope S, Hoseth E, Morch R, Reponen E, et al. Childhood maltreatment severity is associated with elevated C-reactive protein and body mass index in adults with schizophrenia and bipolar diagnoses. Brain Behav Immun. 2017;65:342-9. Epub 2017/06/18. doi: 10.1016/j.bbi.2017.06.005. PubMed PMID: 28619247.

2. Bertone-Johnson ER, Whitcomb BW, Missmer SA, Karlson EW, Rich-Edwards JW. Inflammation and early-life abuse in women. Am J Prev Med. 2012;43(6):611-20. Epub 2012/11/20. doi: 10.1016/j.amepre.2012.08.014. PubMed PMID: 23159256; PubMed Central PMCID: PMCPMC3504353.

3. Corsi-Zuelli F, Loureiro CM, Shuhama R, Fachim HA, Menezes PR, Louzada-Junior P, et al. Cytokine profile in first-episode psychosis, unaffected siblings and community-based controls: the effects of familial liability and childhood maltreatment. Psychol Med. 2019:1-9. Epub 2019/05/09. doi: 10.1017/s0033291719001016. PubMed PMID: 31064423.

4. Counotte J, Bergink V, Pot-Kolder R, Drexhage HA, Hoek HW, Veling W. Inflammatory cytokines and growth factors were not associated with psychosis liability or childhood trauma. PLoS One. 2019;14(7):e0219139. Epub 2019/07/06. doi: 10.1371/journal.pone.0219139. PubMed PMID: 31276524; PubMed Central PMCID: PMCPMC6611659.

5. Danese A, Pariante CM, Caspi A, Taylor A, Poulton R. Childhood maltreatment predicts adult inflammation in a life-course study. Proceedings of the National Academy of Sciences of the United States of America. 2007;104(4):1319-24. doi: <http://dx.doi.org/10.1073/pnas.0610362104>.

6. Dennison U, McKernan D, Cryan J, Dinan T. Schizophrenia patients with a history of childhood trauma have a pro-inflammatory phenotype. Psychol Med. 2012;42(9):1865-71. Epub 2012/02/24. doi: 10.1017/s0033291712000074. PubMed PMID: 22357348.

7. Grassi-Oliveira R, Brietzke E, Pezzi JC, Lopes RP, Teixeira AL, Bauer ME. Increased soluble tumor necrosis factor-alpha receptors in patients with major depressive disorder. Psychiatry Clin Neurosci. 2009;63(2):202-8. Epub 2009/01/30. doi: 10.1111/j.1440-1819.2008.01918.x. PubMed PMID: 19175760.

8. Hartwell KJ, Moran-Santa Maria MM, Twal WO, Shaftman S, DeSantis SM, McRae-Clark AL, et al. Association of elevated cytokines with childhood adversity in a sample of healthy adults. J Psychiatr Res. 2013;47(5):604-10. Epub 2013/02/19. doi: 10.1016/j.jpsychires.2013.01.008. PubMed PMID: 23415658; PubMed Central PMCID: PMCPMC3594625.

9. Imai R, Hori H, Itoh M, Lin M, Niwa M, Ino K, et al. Inflammatory markers and their possible effects on cognitive function in women with posttraumatic stress disorder. J Psychiatr Res. 2018;102:192-200. Epub 2018/04/24. doi: 10.1016/j.jpsychires.2018.04.009. PubMed PMID: 29684628.

10. Kim S, Watt T, Ceballos N, Sharma S. Adverse childhood experiences and neuroinflammatory biomarkers—The role of sex. Stress and Health. 2019;35(4):432-40. doi: 10.1002/smi.2871.

11. Lehto SM, Elomaa AP, Niskanen L, Herzig KH, Tolmunen T, Viinamäki H, et al. Serum adipokine levels in adults with a history of childhood maltreatment. Progress in Neuro-Psychopharmacology and Biological Psychiatry. 2012;37(2):217-21. doi: 10.1016/j.pnpbp.2012.01.016.

12. Lu S, Peng H, Wang L, Vasish S, Zhang Y, Gao W, et al. Elevated specific peripheral cytokines found in major depressive disorder patients with childhood trauma exposure: A cytokine antibody array analysis. Comprehensive Psychiatry. 2013;54(7):953-61. doi: <http://dx.doi.org/10.1016/j.comppsych.2013.03.026>.

13. Pedrotti Moreira F, Wiener CD, Jansen K, Portela LV, Lara DR, Souza LDDM, et al. Childhood trauma and increased peripheral cytokines in young adults with major depressive: Population-based study. Journal of Neuroimmunology. 2018;319:112-6. doi: <http://dx.doi.org/10.1016/j.jneuroim.2018.02.018>.

14. Muller N, Krause D, Barth R, Myint AM, Weidinger E, Stettinger W, et al. Childhood Adversity and Current Stress are related to Pro- and Anti-inflammatory Cytokines in Major Depression. J Affect Disord. 2019;253:270-6. Epub 2019/05/08. doi: 10.1016/j.jad.2019.04.088. PubMed PMID: 31063941.

15. Pinto Pereira SM, Stein Merkin S, Seeman T, Power C. Understanding associations of early-life adversities with mid-life inflammatory profiles: Evidence from the UK and USA. Brain Behav Immun. 2019;78:143-52. Epub 2019/01/27. doi: 10.1016/j.bbi.2019.01.016. PubMed PMID: 30682500; PubMed Central PMCID: PMCPMC6941353.

16. Porcu M, Machado RCBR, Urbano M, Verri WA, Rossaneis AC, Vargas HO, et al. Depressed female smokers have higher levels of soluble tumor necrosis factor receptor 1. Addictive Behaviors Reports. 2018;7:90-5. doi: <http://dx.doi.org/10.1016/j.abrep.2018.03.004>.

17. De Punder K, Entringer S, Heim C, Deuter CE, Otte C, Wingenfeld K, et al. Inflammatory measures in depressed patients with and without a history of adverse childhood experiences. Frontiers in Psychiatry. 2018;9. doi: 10.3389/fpsyt.2018.00610.

18. Slopen N, McLaughlin KA, Dunn EC, Koenen KC. Childhood adversity and cell-mediated immunity in young adulthood: Does type and timing matter? Brain, Behavior, and Immunity. 2013;28:63-71. doi: <http://dx.doi.org/10.1016/j.bbi.2012.10.018>.

19. Smith AK, Conneely KN, Kilaru V, Mercer KB, Weiss TE, Bradley B, et al. Differential immune system DNA methylation and cytokine regulation in post-traumatic stress disorder. American Journal of Medical Genetics, Part B: Neuropsychiatric Genetics. 2011;156(6):700-8. doi: 10.1002/ajmg.b.31212.

20. Thurston RC, Chang Y, Barinas-Mitchell E, Von Känel R, Richard Jennings J, Santoro N, et al. Child abuse and neglect and subclinical cardiovascular disease among midlife women. Psychosomatic Medicine. 2017;79(4):441-9. doi: 10.1097/PSY.0000000000000400.

21. Toft H, Neupane SP, Bramness JG, Tilden T, Wampold BE, Lien L. The effect of trauma and alcohol on the relationship between level of cytokines and depression among patients entering psychiatric treatment. BMC Psychiatry. 2018;18(1):95. Epub 2018/04/11. doi: 10.1186/s12888-018-1677-z. PubMed PMID: 29631540; PubMed Central PMCID: PMCPMC5891976.

22. Waldron JC, Scarpa A, Kim-Spoon J, Coe CL. Adult Sexual Experiences as a Mediator Between Child Abuse and Current Secretory Immunoglobulin A Levels. Journal of interpersonal violence. 2016;31(5):942-60. doi: <http://dx.doi.org/10.1177/0886260514556763>.

23. Zeugmann S, Quante A, Popova-Zeugmann L, Kossler W, Heuser I, Anghelescu I. Pathways linking early life stress, metabolic syndrome, and the inflammatory marker fibrinogen in depressed inpatients. Psychiatr Danub. 2012;24(1):57-65. Epub 2012/03/27. PubMed PMID: 22447087.
