# Supplementary material for: The association of child maltreatment and systemic inflammation in adulthood: A systematic review
Source: PLoS One. 2021 Apr 8;16(4):e0243685. doi: 10.1371/journal.pone.0243685 (PMC8031439; doi:10.1371/journal.pone.0243685)
Supplement: S1 File — (DOCX) [file pone.0243685.s003.docx]

Supplement 1- Search Strategy

PubMed/Medline

 (((((("child abuse"[Title/Abstract] OR "child maltreatment"[Title/Abstract] OR "childhood abuse"[Title/Abstract] OR "childhood maltreatment"[Title/Abstract])) OR ("sexual abuse"[Title/Abstract] OR "physical abuse"[Title/Abstract] OR "emotional abuse"[Title/Abstract] OR "emotional neglect"[Title/Abstract] OR "physical neglect"[Title/Abstract] OR "child neglect"[Title/Abstract] OR "childhood neglect"[Title/Abstract])) OR ("Childhood Adversity"[Title/Abstract] OR "child adversity"[Title/Abstract] OR "early adversity"[Title/Abstract] OR "early life adversity"[Title/Abstract] OR "early life stress"[Title/Abstract] OR "ELS"[Title/Abstract])) OR ("adverse childhood experience$"[Title/Abstract] OR "Child Adverse Events"[Title/Abstract] OR "childhood adverse events"[Title/Abstract])))
n= 33576

OR ((((((((("Child Abuse"[MeSH Terms]) OR "Child abuse, sexual"[MeSH Terms]) OR "adult survivors of child abuse"[MeSH Terms]) OR "physical abuse"[MeSH Terms]) OR "child, abandoned"[MeSH Terms]) OR "adolescent, institutionalized"[MeSH Terms]) OR "adult survivors of child adverse events"[MeSH Terms]) OR "adverse childhood experiences"[MeSH Terms]))
n=33519

Combined Abuse/Neglect term (MESH and title/abstract)= 48144

((((("inflammation"[Title/Abstract] OR "inflammatory"[Title/Abstract] OR "immune"[Title/Abstract] OR "Immunology"[Title/Abstract] OR "Immunological"[Title/Abstract])) OR ("C-Reactive Protein"[Title/Abstract] OR "C Reactive Protein"[Title/Abstract] OR "CRP"[Title/Abstract] OR "Interleukin$"[Title/Abstract] OR "Cytokine$"[Title/Abstract] OR "Tumour Necrosis Factor"[Title/Abstract] OR "TNF"[Title/Abstract] OR "Fibrinogen"[Title/Abstract])) OR ("NF-Kappa B"[Title/Abstract] OR "Nuclear Factor KB"[Title/Abstract] OR "Nuclear Factor Kappa B"[Title/Abstract] OR "Leukocyte$"[Title/Abstract] OR "lymphocyte$"[Title/Abstract] OR "White blood cells"[Title/Abstract] OR "white cell count"[Title/Abstract] OR "acute phase protein$"[Title/Abstract])))

N=1931715

OR (((((((((((inflammation[MeSH Terms]) OR C-Reactive Protein[MeSH Terms]) OR Acute phase proteins[MeSH Terms]) OR Tumour Necrosis Factor Alpha[MeSH Terms]) OR Interleukins[MeSH Terms]) OR cytokines[MeSH Terms]) OR immune system[MeSH Terms]) OR fibrinogen[MeSH Terms]) OR NF-kappa B[MeSH Terms]) OR leukocytes[MeSH Terms]) OR lymphocytes[MeSH Terms])

N=1997818

Combined Immune term= 3010118

Abuse/Neglect AND Inflammation= 977 on 15/5/20

EMBASE

(Child Abuse or Child Abuse, Sexual or Adult Survivors of Child Abuse or Physical Abuse or Sexual abuse or Child, Abandoned or Adolescent, Institutionalized or Adult Survivors of Child Adverse Events or Adverse Childhood Experiences).kw.
n=8929

("Child Abuse" or "Childhood Abuse" or "Sexual Abuse" or "Physical Abuse" or "Emotional Abuse" or "Emotional Neglect" or "Physical Neglect" or "Child Neglect" or "Childhood Neglect" or "Child Maltreaetment" or "Childhood Maltreatment").ti. or ("Child Abuse" or "Childhood Abuse" or "Sexual Abuse" or "Physical Abuse" or "Emotional Abuse" or "Emotional Neglect" or "Physical Neglect" or "Child Neglect" or "Childhood Neglect" or "Child Maltreatment" or "Childhood Maltreatment").ab. or ("Adverse Childhood Experience$" or "Child Adversity" or "Childhood Adversity" or "Child Adversity" or "Early Life Adversity" or "Early Life Stress" or "ELS" or "Childhood Adverse Events" or "Child Adverse Events").ti. or ("Adverse Childhood Experience$" or "Child Adversity" or "Childhood Adversity" or "Child Adversity" or "Early Life Adversity" or "Early Life Stress" or "ELS" or "Childhood Adverse Events" or "Child Adverse Events").ab.
n=34604

Keyword OR Title/Abstract = 36502

(Inflammation or C-Reactive Protein or Acute Phase Proteins or Tumour Necrosis Factor Alpha or Interleukins or Cytokines or Immune System or Fibrinogen or NF-Kappa B or white blood cells).kw.
n=194363

(Inflammation or Inflammatory or Immune or Immunological or Immunology).ti. or (Inflammation or Inflammatory or Immune or Immunological or Immunology).ab. or ("C-Reactive Protein" or "C Reactive Protein" or "CRP" or "Tumour Necrosis Factor" or "TNF" or "TNF-A" or "Interleukin$" or "Cytokine$" or "Fibrinogen" or "NF-Kappa B" or "Nuclear Factor Kappa B" or "Nuclear Factor KB" or "Leukocyte$" or "Lymphocyte$" or "White Blood Cells" or "Acute Phase Protein$").ti. or ("C-Reactive Protein" or "C Reactive Protein" or "CRP" or "Tumour Necrosis Factor" or "TNF" or "TNF-A" or "Interleukin$" or "Cytokine$" or "Fibrinogen" or "NF-Kappa B" or "Nuclear Factor Kappa B" or "Nuclear Factor KB" or "Leukocyte$" or "Lymphocyte$" or "White Blood Cells" or "Acute Phase Protein$").ab.
n=2338153

Keyword OR title/abstract= 2355079

Abuse/Neglect AND Inflammation on 15/5/20= 1336

SCOPUS

KEY ( "Child Abuse"  OR  "Childhood Abuse"  OR  "Sexual Abuse"  OR  "Physical Abuse"  OR  "Emotional Abuse"  OR  "Emotional Neglect"  OR  "Physical Neglect"  OR  "Child Neglect"  OR  "Childhood Neglect"  OR  "Child Maltreaetment"  OR  "Childhood Maltreatment"  OR  "Adverse Childhood Experience$"  OR  "Child Adversity"  OR  "Childhood Adversity"  OR  "Child Adversity"  OR  "Early Life Adversity"  OR  "Early Life Stress"  OR  "ELS"  OR  "Childhood Adverse Events"  OR  "Child Adverse Events" )  AND  KEY ( inflammation  OR  inflammatory  OR  immune  OR  immunological  OR  immunology  OR  "C-Reactive Protein"  OR  "C Reactive Protein"  OR  "CRP"  OR  "Tumour Necrosis Factor"  OR  "TNF"  OR  "TNF-A"  OR  "Interleukin$"  OR  "Cytokine$"  OR  "Fibrinogen"  OR  "NF-Kappa B"  OR  "Nuclear Factor Kappa B"  OR  "Nuclear Factor KB"  OR  "Leukocyte$"  OR  "Lymphocyte$"  OR  "White Blood Cells"  OR  "Acute Phase Protein$" )

N=1739

PsychInfo

KEY ( "Child Abuse"  OR  "Childhood Abuse"  OR  "Sexual Abuse"  OR  "Physical Abuse"  OR  "Emotional Abuse"  OR  "Emotional Neglect"  OR  "Physical Neglect"  OR  "Child Neglect"  OR  "Childhood Neglect"  OR  "Child Maltreaetment"  OR  "Childhood Maltreatment"  OR  "Adverse Childhood Experience$"  OR  "Child Adversity"  OR  "Childhood Adversity"  OR  "Child Adversity"  OR  "Early Life Adversity"  OR  "Early Life Stress"  OR  "ELS"  OR  "Childhood Adverse Events"  OR  "Child Adverse Events" )  AND  KEY ( inflammation  OR  inflammatory  OR  immune  OR  immunological  OR  immunology  OR  "C-Reactive Protein"  OR  "C Reactive Protein"  OR  "CRP"  OR  "Tumour Necrosis Factor"  OR  "TNF"  OR  "TNF-A"  OR  "Interleukin$"  OR  "Cytokine$"  OR  "Fibrinogen"  OR  "NF-Kappa B"  OR  "Nuclear Factor Kappa B"  OR  "Nuclear Factor KB"  OR  "Leukocyte$"  OR  "Lymphocyte$"  OR  "White Blood Cells"  OR  "Acute Phase Protein$" )

n=294
