# Supplementary material for: The association of child maltreatment and systemic inflammation in adulthood: A systematic review
Source: PLoS One. 2021 Apr 8;16(4):e0243685. doi: 10.1371/journal.pone.0243685 (PMC8031439; doi:10.1371/journal.pone.0243685)
Supplement: S3 File — (PDF) [file pone.0243685.s005.pdf]

Systematic Review Protocol:

Title: Childhood abuse and neglect and inflammation in adulthood: a systematic review and meta-analysis

Review Team: Dr Daniel Kerr, Prof Helen Minnis

Institution: Institute of Health and Wellbeing, University of Glasgow

Contact: Daniel.Kerr@glasgow.ac.uk

Date of Protocol: 5/6/20

## 1. Background to Review

Childhood maltreatment is common worldwide[12]. Studies have consistently shown childhood maltreatment, particularly multiple and cumulative exposures, to be associated with a range of adverse physical, psychological, and social outcomes in adolescence and adulthood[1,3–5]. That this association persists after adjustment for environmental and behavioural factors suggests underlying biological mechanisms which mediate the relationship between childhood maltreatment and health and social outcomes in later life[6,78]. Understanding the biological correlates of maltreatment will help to clarify the mechanisms linking maltreatment with adverse outcomes; and offers the prospect of enhanced risk stratification of young people who have been subject to maltreatment; and may identify new treatment targets to break the link between childhood experiences and adverse physical and mental health outcomes in adulthood [2,910].

A range of biomarkers associated with childhood maltreatment have been identified and offer the beginnings of a unified model linking childhood maltreatment with adult health. These include neurobiological changes, epigenetics, endocrine dysfunction, and inflammation[6,11–13].

Low-grade inflammation is generally defined as 2-3 fold elevations in inflammatory markers like CRP, Interleukin-6 and TNF-a[17]. This represents a chronic low-level activation of the immune system (likely representing excessive sensitivity to inflammatory stimuli and deficiencies of the anti-inflammatory pathways which would normally terminate such responses) and is distinguished from high grade inflammatory states with markedly elevated inflammatory markers like acute infections, severe illnesses, and auto-inflammatory diseases. Low-grade inflammation has been associated with a range of health conditions such as cardiovascular disease and diabetes[18,19]. Notably a large body of work physical has associated low-grade elevations in CRP (as measured by highly sensitive assay-hsCRP) with cardiovascular events however subsequent work has questioned the causality of this relationship[18]. Other inflammatory markers have been associated with cardiovascular disease particularly Interleukin-6. A large international study using Mendellian Randomisation techniques has supported a causal relationship between elevated levels of Il-6 and heart disease[19]. Further supporting evidence for the role of low-grade inflammation in cardiovascular disease is provided by the CANTO trial of the specific Il-1b antagonist Canakinumab which was shown to reduce rates of myocardial infarction, stroke and death in patients treated following an MI with elevated hsCRP[20]. Low-grade inflammation is also associated with a range mental health disorders. A wide body of work has associated major depressive disorder with low-grade elevations in inflammatory markers like CRP, Il-6, and TNF-a[21]. The neurobiological effects of peripheral cytokines may mediate the relationship between external stressors and depression[21]. Low-grade inflammation is also associated with conditions like Post-Traumatic Stress Disorder[17], Schizophrenia, and Bipolar Affective Disorder[12,22]. Interestingly a recent Mendellian Randomisation analysis have suggested a causal relationship of CRP with schizophrenia and bipolar affective disorder[22].

Low-grade inflammation is significantly associated with major physical and mental health disorders, and an emerging body of evidence suggests that this relationship is at least partially causal. Low-grade inflammation has been identified in adult survivors of childhood maltreatment. Previous reviews have identified significant heterogeneity in the literature particularly in relation to the definition and assessment of childhood maltreatment[23,24]. Studies have offered varying definitions of childhood maltreatment ranging from narrowly focused childhood physical or sexual abuse, to more broadly defined ACEs, and wider environmental stressors such as poverty and natural disasters. It is likely that different patterns of maltreatment will have different effects on development and so wide definitions introduce significant heterogeneity. In particular it is emerging that consequences of deprivation/neglect differ substantially from active abuse and may lead to different biological sequelae[2] In this light this review aims to reduce heterogeneity by limiting its scope to the association of low-grade inflammation with childhood abuse or neglect.

References:


1. Gilbert, R. *et al.* Burden and consequences of child maltreatment in high-income countries. *Lancet* **373,** 68–81 (2009).

2. Nemeroff, C. B. Paradise Lost: The Neurobiological and Clinical Consequences of Child Abuse and Neglect. *Neuron* **89,** 892–909 (2016).

3. Hughes, K. *et al.* The effect of multiple adverse childhood experiences on health: a systematic review and meta-analysis. *Lancet Public Heal.* **2,** e356–e366 (2017).

4. Anda, R. F. *et al.* Household Dysfunction to Many of the Leading Causes of Death in Adults The Adverse Childhood Experiences ( ACE ) Study. *Am. J. Prev. Med. 0749-3797/98/$19.00* **14,** 245–258 (1998).

5. S.R., D., V.J., F., M., D. & W.H., G. The impact of adverse childhood experiences on health problems: Evidence from four birth cohorts dating back to 1900. *Prev. Med. (Baltim).* **37,** 268–277 (2003).

6. Nemeroff, C. Neurobiology of gene-environment interactions in mediating child abuse associated risk for mood and anxiety disorders. *Neuropsychopharmacology* **36,** S48–S49 (2011).

7. Danese, A. *et al.* Biological embedding of stress through inflammation processes in childhood. *Mol. Psychiatry* **16,** 244–246 (2011).

8. Oh, D. L. *et al.* Systematic review of pediatric health outcomes associated with childhood adversity. *BMC Pediatr.* **18,** 83 (2018).

9. Nemeroff, C. Neurobiological consequences of child abuse. *Int. J. Neuropsychopharmacol.* **17,** 24 (2014).

10. Gilmer, W. S. *et al.* Ameliorating the biological impacts of childhood adversity: A review of intervention programs. *Child Abus. Negl.* **81,** 82–105 (2018).

11. York, T. *et al.* Increased chromosomal damage in adults with a history of childhood sexual abuse: Moving toward a model of biological mechanism. *Behav. Genet.* **42,** 977–978 (2012).

12. Biological pathways between childhood trauma and psychosis onset. *Eur. Arch. Psychiatry Clin. Neurosci.* **263,** S38–S39 (2013).

13. A., D. Adverse childhood experiences, allostasis, allostatic load, and age-related disease. *Physiol. Behav.* **106,** 29–39 (2012).

14. Szyf, M. Examining peripheral DNA methylation in behavioral epigenetic and epigenetic psychiatry: Opportunities and challenges. *Epigenomics* **6,** 581–584 (2014).

15. Mondelli, V. From childhood trauma to psychosis onset: The role of HPA axis and inflammation. *Biol. Psychiatry* **77,** 324S (2015).

16. Mondelli, V. Hypothalamus-pituitary-adrenal (HPA) axis and inflammation as mediators of the association between childhood trauma and onset of psychosis. *Neuropsychopharmacology* **39,** S100 (2014).

17. Speer, K., Upton, D., Semple, S. & McKune, A. Systemic low-grade inflammation in post-traumatic stress disorder: A systematic review. *J. Inflamm. Res.* **11,** 111–121 (2018).

18. Protein, R., Heart, C. & Genetics, D. Association between C reactive protein and coronary heart disease: mendelian randomisation analysis based on individual participant data. *Bmj* **342,** d548–d548 (2011).

19. Sarwar, N. *et al.* Interleukin-6 receptor pathways in coronary heart disease: A collaborative meta-analysis of 82 studies. *Lancet* **379,** 1205–1213 (2012).

20. Ridker, P. M. *et al.* Antiinflammatory Therapy with Canakinumab for Atherosclerotic Disease. *N. Engl. J. Med.* NEJMoa1707914 (2017). doi:10.1056/NEJMoa1707914

21. Dantzer, R., Connor, J. C. O., Freund, G. G., Johnson, R. W. & Kelley, K. W. L 0.8 l. **9,** 8 (2010).

22. Ligthart, S. *et al.* Genome Analyses of >200,000 Individuals Identify 58 Loci for Chronic Inflammation and Highlight Pathways that Link Inflammation and Complex Disorders. *Am. J. Hum. Genet.* **103,** 691–706 (2018).

23. Slopen, N., Koenen, K. C. & Kubzansky, L. D. Childhood adversity and immune and inflammatory biomarkers associated with cardiovascular risk in youth: A systematic review. *Brain. Behav. Immun.* **26,** 239–250 (2012).

24. R., C., T.W., V., C., W.-B. & E., B. Childhood maltreatment and inflammatory markers: A systematic review. *Acta Psychiatr. Scand.* **129,** 180–192 (2014).

## 2. Research Questions:

**Primary research question:**
Is exposure to abuse and/or neglect in childhood associated with elevated markers of inflammation in later life (>18s).

**Secondary Research Questions:**
On the basis of the current literature can we comment on the following questions:
- Is there differences in patterns of later life inflammation associated with specific types, timings or duration of abuse?
- What mediates any association between childhood abuse and neglect and inflammation in later life? (In particular how much of this association is mediated by BMI?)

3. Criteria for including studies in the review

| | |
|---|---|
| Population | **Inclusion:**<br>1) Inflammatory marker measured in adulthood (>18 years of age). No upper age limits.<br>2) Includes participants exposed to abuse/neglect and controls OR a continuous measure of abuse/neglect exposure<br><br>**Exclusion:**<br>1) <18s<br>2) Pro-inflammatory physical health states (eg. cancer, auto-immune disease)<br>3) Animal studies |
| Exposure | -Exposure to physical abuse, sexual abuse, emotional abuse, physical neglect and/or emotional neglect occurring before the age of 18.<br><br>-Studies may specify a form of abuse or neglect or report an overall abuse/neglect outcome.<br><br>- Exposure may be recorded prospectively and retrospectively.<br><br>- No specific recording tool is required but studies must specify how abuse/neglect status was ascertained.<br><br>- Studies may compare a group exposed to abuse/neglect with an unexposed control group OR utilise a continuous measure of abuse/neglect exposure in the total sample (eg. Childhood Trauma Questionnaire as a continuous measure).<br><br>- Wider adverse childhood experiences (eg. parental mental illness, parental separation, parental imprisonment, exposure to domestic violence, bullying etc) are excluded as the study is specifically focused on abuse/neglect. Studies of ACEs which report specific effects of abuse and neglect will be included, but studies which solely report on overall ACEs scores will be excluded. |
| Comparison | Studies may compare a group exposed to abuse and/or neglect with an unexposed control group OR utilise a continuous measure of abuse/neglect exposure in the total sample (eg. Childhood Trauma Questionnaire as a continuous measure). |
| Outcome | Blood levels of inflammatory markers. Any marker of the inflammatory response measured in the blood is eligible for inclusion.<br>**Exclusion**:<br>- Studies exclusively reported on stimulated response of inflammatory markers (eg.to social stressors or biological stimulation) |

| | |
|---|---|
| | - Studies exclusively reporting on gene expression<br>- Studies exclusively reporting on *in vitro* production of inflammatory markers by immune cells<br>- Studies exclusively measuring inflammation in the central nervous system (eg. CSF) |
| Setting | Relevant studies may involve hospitalised patients, outpatients, or participants recruited from the general population. There is no restriction by country of origin. |
| Study Design | Non-randomised observational studies. Retrospective or prospective. |

Inclusion and Exclusion Criteria for Studies

**Inclusion:**

1) Non-randomised observational studies. Both retrospective and prospective studies are eligible for inclusion.
2) Compares blood levels of an inflammatory marker between an abuse/neglect exposed group and an unexposed control group OR examine the association of continuous measures of abuse/neglect exposure and inflammatory markers in the total sample.
3) Inflammatory markers measured in adulthood (>18)

Exclusion:
1) Participants <18
2) Studies not measuring blood levels of an inflammatory marker (eg. studies reporting expression of inflammation related genes, in vitro production of inflammatory cytokines by immune cells, CSF)
3) Studies exclusively reporting on stimulated production of inflammatory markers (eg. in response to stress testing or biological immune stimulation)
4) Studies of populations with inflammatory physical conditions (eg. cancer)
5) Animal studies

4. Search Methods:

| | |
|---|---|
| Electronic Databases | Pubmed/Medline<br>Embase<br>PsychInfo<br>Scopus |
| Other methods used for identifying relevant research | Reference checking and hand searching of these.<br>References of previous reviews in this area. |

## 5. Methods of Review:

| | |
|---|---|
| Details of Methods | 1 reviewer will screen records against inclusion criteria. A 2nd reviewer will review eligible articles to confirm that they meet inclusion criteria. Disagreements will be resolved by conference.<br>Mendeley will be used to keep track of references. |
| Quality Assessment | Risk of bias assessment at study level. Formal assessment of risk of bias using the Crowe Critical Appraisal Tool v1.4 (https://conchra.com.au/wp-content/uploads/2015/12/CCAT-form-v1.4.pdf). This is a tool for assessment of risk of bias in non-randomised studies. Key components of risk of bias assessment in sampling, ascertainment of exposure, measurement of outcome, and statistical analysis including adjustment for relevant confounding variables. The CCAT assigns a total score from 0-40. According to the tools guidelines this can be categorised as low quality (<20), moderate quality (20-29), and high quality (30+).<br>1 reviewer will rate all papers. A 2nd reviewer will co-rate a sub-sample of 25% of articles. If agreement is <90% all papers will be co-rated. Any disagreements will be resolved by conference.<br>Quality of included articles will be presented in the final article. In meta-analysis risk of bias will be included in sensitivity analyses. |
| Data Extraction | A standardised data extraction form has been developed (using word). Extracted data:<br><br>- Reference<br>- Sample size<br>- Age (mean and SD preferred)<br>- Gender (no. and % of female participants)<br>- BMI (mean and SD preferred)<br>- Study setting (eg. hospital, outpatient clinic, college, community)<br>- Population type (eg. clinical- mental and/or physical illness, general population)<br>- Study type (prospective or retrospective)<br>- Measure of abuse<br>- Severity of abuse in sample (for studies using continuous scales)<br>- Inflammatory markers measured<br>- Details of assay used<br>- Statistical techniques used for analysis<br>- Variables controlled for in analysis (if relevant)<br>- Results<br>- Funding details<br>- Free text for additional limitations or other points. |
| Narrative Synthesis | Narrative synthesis will be developed alongside meta-analysis. Key areas to explore will include:<br>- Identification of any gaps in the literature<br>- Identification of methodological limitations in the literature<br>- Exploration of the role of confounding factors (such as BMI) on the association between abuse and neglect and inflammation<br>- Discussion of findings relating to inflammatory markers which are measured in insufficient studies to perform meta-analysis. |
| Meta-analysis | We aim to perform meta-analysis for inflammatory markers with >5 studies reporting results.<br>- Heterogenity will be assessed when data is available, it is likely that a random effects meta-analysis will be most appropriate.<br>- Scoping review indicates that outcomes are analysed and reported in an inconsistent manner (eg. difference in means, linear regression coefficients, hazard ratios). For purpose of analysis we would aim to present findings using standardised effect sizes.<br><br>Additional Analyses |

| | |
|---|---|
| | Depending on data reporting, we would aim how factors influence the relationship between abuse and neglect and inflammation and perform meta-regression analysis if possible. Based on scoping review we pre-specify the following variables for sub-group analysis if allowed by the data:<br><br>1) Retrospective vs prospective studies: Previous work has suggested that prospective and retrospective measures of abuse capture different populations. Significant differences in findings based on methodologies would be a significant finding informing future research in this area.<br>2) Clinical vs non-clinical samples: It would inform our understanding of risk stratification and possible mechanisms if an association differed between clinical and non-clinical samples.<br>3) Abuse sub-types: Some studies have suggested that there are different effects for different types of abuse, particularly childhood sexual abuse.<br>4) BMI: studies have identified varying results on the influence of BMI, notably some studies suggest that BMI may largely or entirely mediate the association of abuse/neglect and inflammation in later life.<br>5) Gender: Some studies have suggested a gender impact wherein the effect is greater in females but this is not a universal finding. |

## 6. Dissemination of Results:

We aim to publish our completed review in a high impact journal and present at a relevant international conference.
